# Assessing disparities in cancer resources distribution in Mexico

**DOI:** 10.1186/s12913-025-12497-z

**Published:** 2025-04-17

**Authors:** Elysse Bautista-Gonzalez, Andrés Quintero Leyra, Teresa Verenice Munoz Rocha, Heber Tomás Reyes-García, Enrique Soto-Perez-de-Celis, Alejandra Palafox Parrilla, Alejandro Mohar Betancourt, Richard Sullivan

**Affiliations:** 1 Fundación Mexicana para la salud, Anillo Perif. 4809, Arenal Tepepan, Tlalpan, Ciudad de México, 14610 México; 2 Institute of Epidemiology and Health care, University College London, 1-19 Torrington Pl, London, WC1E 7HB United Kingdom; 3 Public Health Department, Faculty of Medicine, National Autonomous University of Mexico, Av. Universidad 3004, Copilco Universidad, Coyoacán, Ciudad de México, CDMX, 04510 México; 4 Centro de Investigación en Nutrición y Salud, Instituto Nacional de Salud Pública, Av. Universidad 655, Santa María Ahuacatitlán, Cuernavaca, Mor 62100 México; 5 Centro Médico Nacional 20 de Noviembre, Félix Cuevas 540, Col del Valle Sur, Benito Juárez, Ciudad de México, CDMX, 03104 México; 6 Division of Medical Oncology, University of Colorado Anschutz Medical Campus, 12801 East 17th Avenue, 8122, Aurora, CO 80045 USA; 7 Unidad de Epidemiología e Investigación Biomédica en Cáncer, Instituto Nacional de Cancerología, Av. San Fernando 22, Belisario Domínguez Secc 16, Tlalpan, Ciudad de México, CDMX, 14080 México; 8 Unidad de Investigación Biomédica en Cáncer, Instituto Nacional de Cancerología e Instituto de Investigaciones Biomédicas, UNAM, Av. Universidad 3004, Copilco Universidad, Coyoacán, Ciudad de México, CDMX, 04510 México; 9 Institute of Cancer Policy, King’s College London, Guy’s Hospital Campus, London, SE1 9RT UK

**Keywords:** Cancer, Infrastructure, Health services research, Low-middle-income country, Mexico, Cáncer, Infraestructura, Investigación en servicios de salud, México

## Abstract

**Background:**

Given the rising cancer burden, the capacity of Mexico’s healthcare system to effectively address cancer care through its current available infrastructure becomes increasingly critical. Limited availability of diagnostic and therapeutic infrastructure leads to delays in diagnosis and treatment. Countries like Mexico, should undertake comprehensive assessments of infrastructure and human resources available for cancer, including its quantification and geolocation, to understand the service gaps. This study seeks to map oncological infrastructure in Mexico in five types of cancer: breast, lung, prostate, colon, and cervix.

**Methods:**

Through a realist evaluation of publicly available databases from the *High Specialty Medical Equipment National Inventory* and the *General Direction of Health Information*, a comprehensive identification and classification of cancer resources was conducted with the intended outcome to map cancer care infrastructure in Mexico. Guided by the literature, resources necessary for diagnosis and treatment were selected by an expert consensus. Thereafter, facilities were classified by *service scope* into either diagnostic or diagnostic and therapeutic, and by *infrastructure level* into core or enhanced and then mapped geographically.

**Results:**

From *N* = 14,133 unique healthcare facilities that deliver any type of healthcare, only 5% provided cancer care. Cancer-specific infrastructure that can provide diagnosis and treatment at the national level included *N* = 10 brachytherapy, *N* = 11 cobalt-60, *N* = 21 linear particle accelerators and *N* = 188 operating rooms. Five issues were found: (1) low availability of core therapeutic infrastructure across all cancer types; (2) regional and national centralization of available therapeutic infrastructure for all cancer types, whilst no centralization found in diagnostic resources; (3) inconsistent allocation of resources in densely populated areas; (4) infrastructure disparities per cancer type i.e., Lung, prostate, and breast cancer require significant investments in diagnostic infrastructure compared to cervical and colon cancer, and (5) lack of precise and updated infrastructure data from the public health system that requires either new codes or subcodes.

**Conclusions:**

Addressing disparities in cancer resources distribution in Mexico is a dual imperative—ensuring equity while seizing an opportunity to fortify the overall health system for people without social security coverage.

**Supplementary Information:**

The online version contains supplementary material available at 10.1186/s12913-025-12497-z.

## Introduction

Cancer has emerged as a significant public health challenge in Mexico, currently ranking as the third leading cause of death in the country [1]. Between 2000 and 2013, cancer accounted for approximately 13% of all deaths, a proportion that remains lower than in developed nations but is steadily rising [1]. Of particular concern is the impact on the economically active population, with 45.4% of cancer-related deaths occurring among individuals aged 15 to 64 years [1]. This burden is exacerbated by high prevalence of risk factors such as smoking, alcohol consumption, excess weight, and obesity, all of which are prevalent in Mexico [1]. Lung, gastric, liver, prostate, breast, and cervical cancers represent the leading causes of cancer-related mortality, together contributing to nearly half of all cancer deaths [1]. Given the rising cancer burden, the capacity of Mexico’s healthcare system to effectively address cancer care through its current available infrastructure becomes increasingly critical [2].

In Mexico there are three main health care providers, those that provide private care, public social security institutions that provide care to the formally employed and the ministry of health that provides care to the otherwise uninsured, unemployed or informally employed [3–5]. Each one of these providers has the governance over what diseases are covered and how resources are allocated and ultimately distributed [3, 5–7]. Moreover, the Mexican healthcare system suffers from the paradox of over-centralization, fragmentation and lack of universal health coverage [3, 8, 9], resulting in unequal access to cancer screening, diagnosis or treatment according to cancer type and affiliation [3–9].

Although macroeconomic factors influence the proportion of the gross domestic product allocated to healthcare, they can sometimes restrict the development of healthcare infrastructure and the recruitment of healthcare personnel [8, 10]. In 2022, Mexico allocated 5.5% of its GDP to healthcare, which is below the Organisation for Economic Co-operation and Development (OECD) average [11]. This limited allocation is reflected in the country’s healthcare infrastructure, where the number of hospital beds per 1000 inhabitants in Mexico is also significantly lower than the OECD average of 4.8, standing at a mere 1.5 [12–16]. Additionally, Mexico lags OECD benchmarks in healthcare personnel, with only 2.5 doctors and 2.9 nurses per 1000 inhabitants [16]. These figures are similar to those of other middle-income countries in the OECD such as Turkey and Brazil, yet they fall short when compared to high-income countries like Finland with nearly seven times more healthcare professionals per capita [16]. This contrast underscores the significant healthcare workforce capacity gap between Mexico and high-income nations.

Specifically for cancer care, the number of specialists is well below the internationally recommended level and insufficient to meet the country’s healthcare needs (20 full time oncologists per million habitants) [17, 18]. One study conducted in 2018 measured the number of specialists per state and found there were 0.71 oncology surgeons, 0.6 paediatric oncology surgeons, 0.31 oncologists, and 0.29 gynaecologist-oncologists, a total of 0.18 non-specific oncologists per million habitants [17]; all found to be concentrated in urban areas, particularly in the three largest cities in Mexico [17]. Moreover, studies looking at cancer related infrastructure have also found significant deficits, as well as concentrations in urban settings, in close proximity to high socio-economic populations [2, 19, 20].

Mapping healthcare resources (or the lack of) has proven to facilitate policy-making for more equitable [8] resource allocation in other contexts [21–24]. However, the public healthcare system covering the uninsured population in Mexico has simply quantified each individual resource (i.e. number of Computed Tomography or CT scans per region, number of specialists) but has not geographically mapped resources considering all the patient needs across the cancer continuum or journey (i.e. laboratory, CT scan, nurses, oncologists, pathologist, nutritionist, etc.) nor has it cross-mapped resources with population density. This study seeks to understand existing gaps in cancer resources distribution in Mexico, particularly in health facilities serving the uninsured population, with the goal of informing policy and advocating for healthcare infrastructure improvements within the Ministry of Health. In alignment with the United Nations Sustainable Development Goals (SDGs), the project supports SDG 3 (Good Health and Well-being) by promoting equitable access to cancer care, SDG 10 (Reduced Inequalities) by addressing regional and population disparities, and SDG 9 (Industry, Innovation, and Infrastructure) by advocating for the development of resilient and accessible health infrastructure.

## Methods

### Data source and eligibility

As part of the Código Cáncer project [25], five site specific cancers were chosen for this cross sectional study, due to high mortality, high incidence-mortality-ratios or impact as catastrophic expenditures [1, 8, 26–31]. Thus, this analysis identifies the available diagnostic and therapeutic infrastructure for five major cancer types in Mexico: breast, lung, prostate, colorectal, and cervical cancers [25].

Publicly available datasets were sought and selected based on their potential to provide comprehensive information on oncological infrastructure, including facilities, equipment, and services for the public healthcare facilities for non-insured patients. These were accessible through official health-related websites in Mexico i.e. from the General Direction of Health Information (DGIS), Health Sectorial Resources 2022 and from the National Centre of Excellence Technology in Health (CENETEC-Salud), the *High Specialty Medical Equipment National Inventory* (EMAT) 2016 [32, 33]. Facilities in Mexico were identified through the “Clave Unica de Establecimientos de Salud” (CLUES). Datasets were merged and in the case of duplication of facilities the most recent resources were considered for the analysis.

### Data classification

This study employed a realist evaluation framework to understand the distribution of cancer infrastructure in Mexico [34]. The context included understanding broader conditions such as the Mexican healthcare system, particularly the resources found in the datasets, and cancer resources needed for diagnosis and treatment according to the clinical guidelines. The mechanism involved categorizing and selecting infrastructure and human resources per cancer type to create a comprehensive classification of resources, and the intended outcome was to map cancer care infrastructure in Mexico.

Six domain experts in oncology and public health in Mexico were involved in initially identifying the diagnostic and therapeutic resources needed by each cancer type (3 oncologists, 1 pathologist, 2 public health researchers). Infrastructure and human resources were categorized and selected per cancer based on existing guidelines [35–43] and on the National Comprehensive Cancer Network Guidelines for resource stratification [44]. Additionally, as part of a realist evaluation [34], to ensure that these were categorised accurately for the Mexican context, consensus was made between the experts to develop a classification based on the available data. For instance, up to the point of cancer suspicion, facilities with either a general practitioner or a specialist (urologist, gynaecologist, gastroenterologist or pneumologist) was considered to be sufficient for diagnostic service scope. However, for the facilities providing both diagnosis and treatment, it was necessary to have a general medical oncologist in the facility to be included in the map, without the necessity of an existing general practitioner. Thus, to avoid over-exclusion of facilities in the maps, experts did not consider general practitioners as an exclusion criterion for facilities marked as diagnostic and therapeutic. It was assumed that in most specialised hospitals providing treatment, which focus on specific diseases like cancer, would not typically employ general practitioners, instead, these hospitals would rely on specialists and subspecialists for patient care.

Furthermore, when a specific imaging technique such as ultrasound was required for diagnosis (but unavailable), higher-technology alternatives like tomography (CT or MRI scans) were used to categorise the facility instead. This ensured that our classification was not overly strict. For example, some breast or colon cancer diagnostic procedures could potentially be done using ultrasound but, if unavailable, they can be done using CT instead. Thus, in such cases, facilities were marked as holding the resource if either one was available.

As a result, two resource categories arose from consensus: *service scope* (diagnostic or diagnostic and therapeutic) and *infrastructure level* (core or enhanced). Table 1 shows the resources available from each dataset and how each one was classified by experts in both service scope and infrastructure level for each cancer type. If deemed unnecessary for the cancer type in question, the cell was coloured in grey. The designation “INDISP” was used to classify each resource when they were indispensable for core patient care and alternatively, the “EXPEND” designation was used when resources would enhance patient care. The rationale for this “EXPEND” was to include facilities that may provide enhanced cancer care through physical therapy, nutritionists or other infrastructure alternatives in addition to diagnosis or treatment.

**Table 1:**
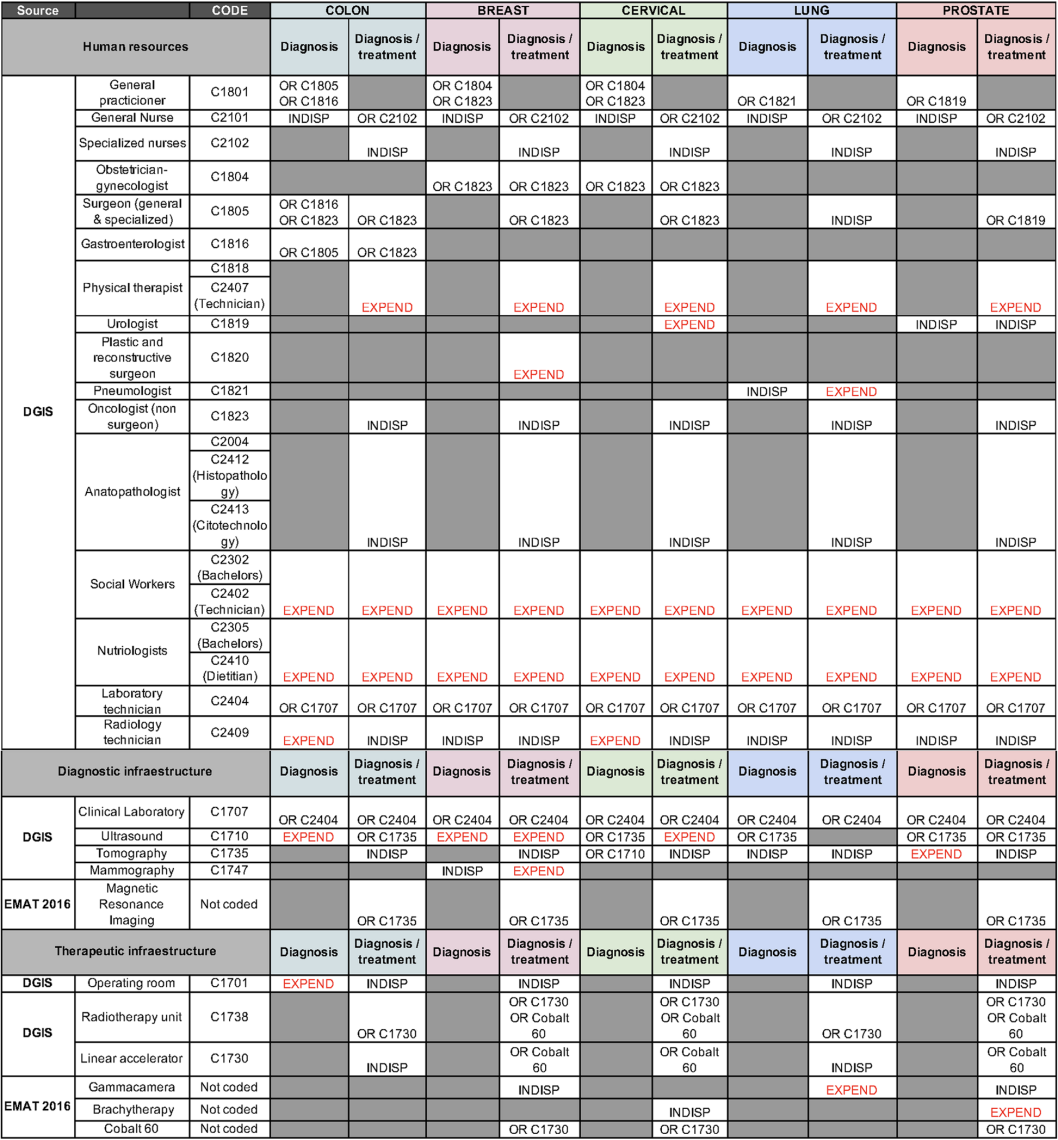
Codes and resource selection per cancer type for service scope and infrastructure level classification

The gray cells were not included for the classification of facilities due to it being unrelated or not relevant to the cancer type at the diagnostic or therapeutic level

### Data mapping

**Fig. 1:**
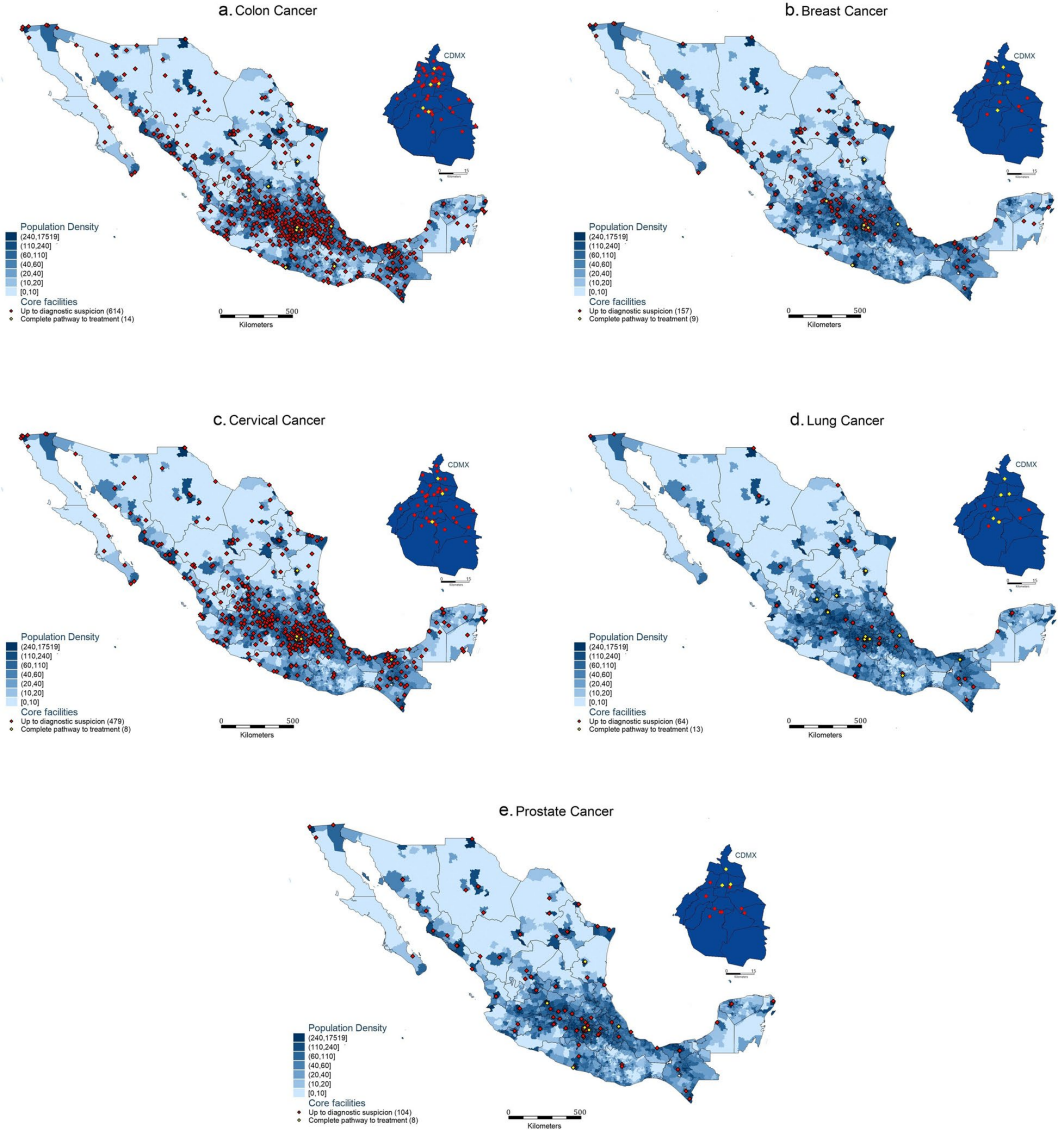
**a**-**e** Geographical distribution of core diagnostic and therapeutic infrastructure for five cancer types in Mexico

**Fig. 2:**
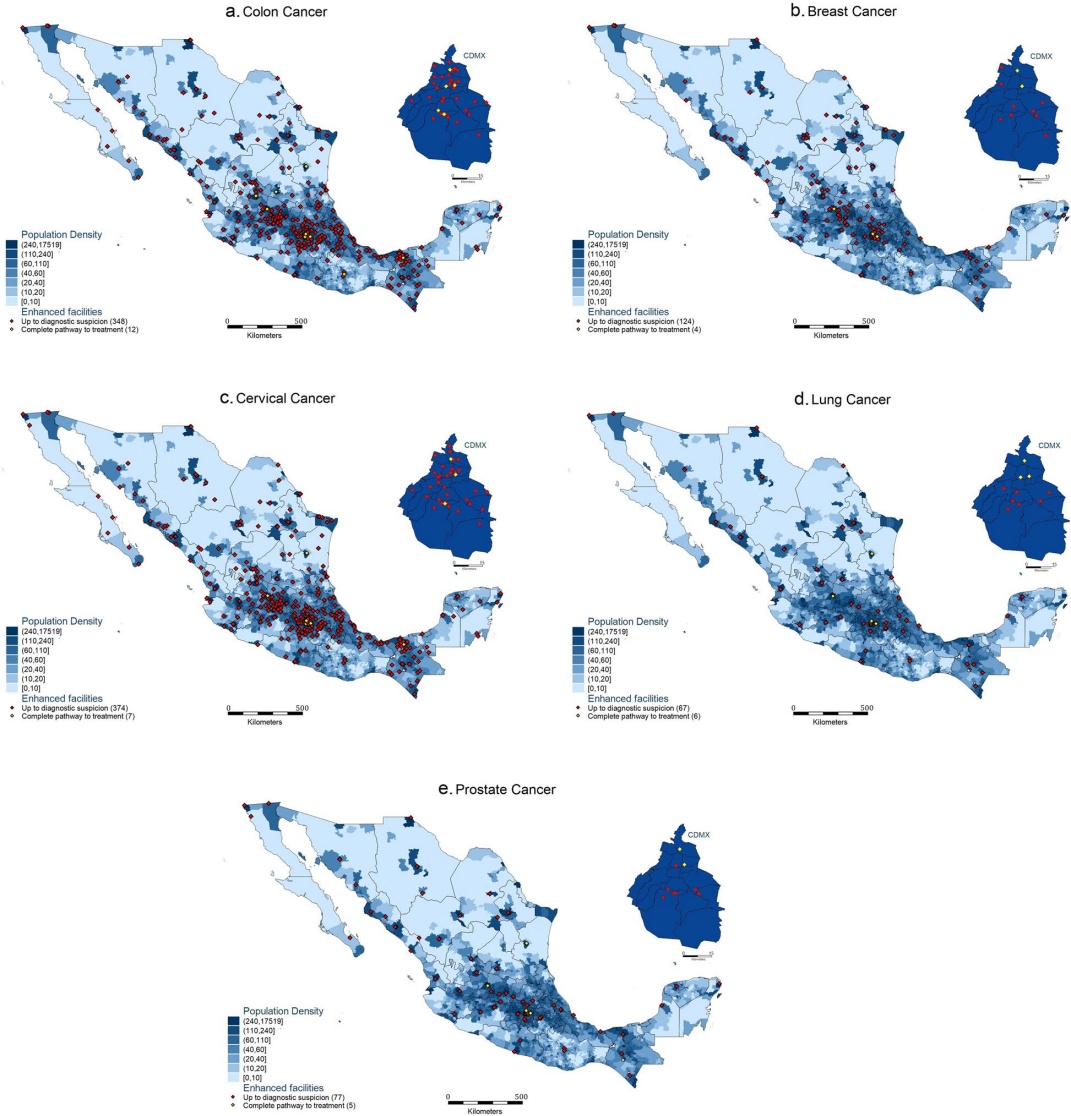
**a**-**e** Geographical distribution of enhanced diagnostic and therapeutic infrastructure for five cancer types in Mexico

Once consensus was reached over the classification of resources, facilities were judged in *service scope* and *infrastructure level*. To be classified into these accordingly, all resources from Table 1 marked in each column as necessary for cancer care, had to be available in the database. As a result of this exercise, facilities were mapped per cancer type according to *service scope* (diagnostic or diagnostic and therapeutic) and *infrastructure level* (core or enhanced). The facilities were mapped against municipal population density to provide visual aid on the geographical distribution of facilities by population. Table 2 describes the two types of maps developed: maps showing facilities providing core cancer care (Fig. 1a-e) and maps showing facilities providing *enhanced care* (Fig. 2a-e).

**Table 2:**
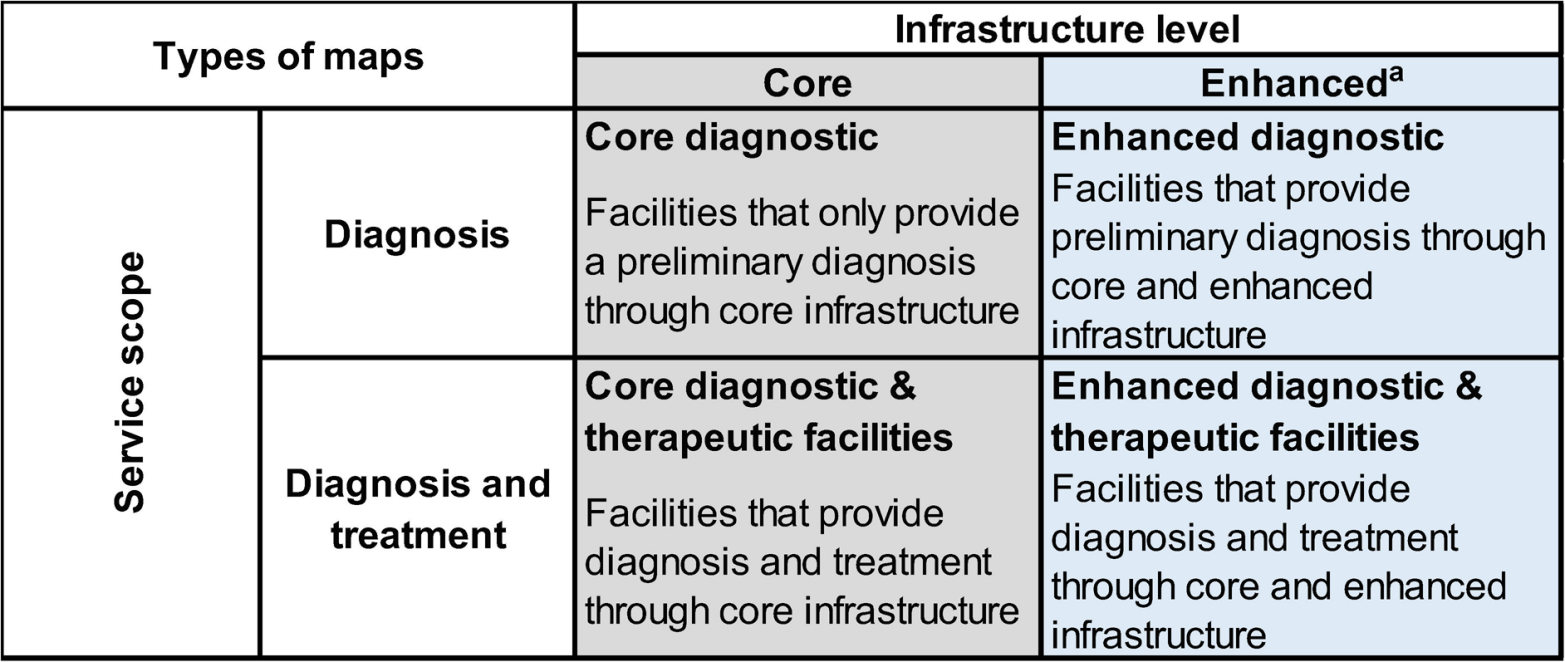
Matrix of types of maps developed through the service scope and infrastructure level classification proposed

^a^The enhance category is similar to the definitions used by NCI

### Data validation

A systematic validation of maps was conducted, involving the revision of a random sample of 25% of the facilities coded resources, and facility verification with the expert panel (i.e. when facilities were known by experts to have oncological resources but did not appear in the map, the team verified the resources preventing the facility to be visible in the map), without finding any misclassifications in either the random sample or the manually searched facilities.

## Results

From our database, a total of 14,133 unique healthcare facilities that deliver any type of healthcare were identified, out of which *N* = 686 (5%) provided cancer care. Table 3 shows the *N* = 686 facilities found stratified by *infrastructure level* (core or enhanced) and *service scope* (diagnostic or diagnostic and therapeutic) split by cancer type.

**Table 3:**
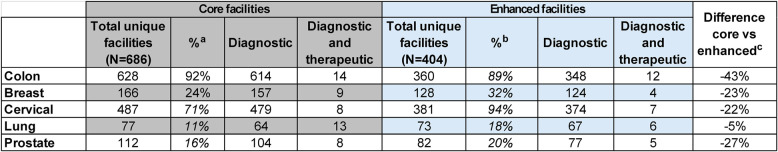
Cancer care facilities identified and mapped by service scope and infrastructure level in Mexico for five cancer types in Mexico

Some facilities could be accounted for multiple times if they were scored as core or enhanced for each cancer type

^a^Percentage of the total number of unique facilities providing core cancer care (*N* = 686)

^b^Percentage of the total number of unique facilities providing enhanced cancer care (*N* = 404)

^c^The relative difference between the total number of core facilities and the total number of enhanced facilities

### Data mapping

Once consensus was reached over the classification of resources, facilities were judged in *service scope* and *infrastructure level*. To be classified into these accordingly, all resources from Table 1 marked in each column as necessary had to be available in the database. As a result of this exercise, facilities were mapped per cancer type according to *service scope* (diagnostic or diagnostic and therapeutic) and *infrastructure level* (core or enhanced). The facilities were mapped against municipal population density to provide visual aid on the geographical distribution of facilities by population. Table 2 describes the two types of maps developed: maps showing facilities providing core cancer care (Fig. 1a-e) and maps showing facilities providing *enhanced care* (Fig. 2a-e).

### Data validation

A systematic validation of maps was conducted, involving the revision of a random sample of 25% of the facilities coded resources, and facility verification with the expert panel (i.e. when facilities were known by experts to have oncological resources but did not appear in the map, the team verified the resources preventing the facility to be visible in the map), without finding any misclassifications in either the random sample or the manually searched facilities.

## Results

From our database, a total of 14,133 unique healthcare facilities that deliver any type of healthcare were identified, out of which *N* = 686 (5%) provided cancer care. Table 3 shows the *N* = 686 facilities found stratified by *infrastructure level* (core or enhanced) and *service scope* (diagnostic or diagnostic and therapeutic) split by cancer type.

### Facilities providing core cancer care

Out of the *N* = 686 facilities providing some type of cancer care, for colon cancer particularly *N* = 628 provided core care (92%). However, only *N* = 14 of those core facilities provided diagnosis and treatment, whilst the rest (*N* = 614) only provided diagnosis. This indicates a significant gap in the availability of core therapeutic options for colon cancer patients. The other types of cancer show similar core infrastructure gaps, cervical cancer facilities totalled 487 facilities (71%), with 479 focusing solely on diagnosis and 8 offering treatment. Accounting for 24% of the total number of cancer facilities, 166 facilities were identified for breast cancer, with 157 offering diagnostic services and only 9 providing both diagnosis and therapeutic care. Lung cancer facilities numbered 77 (11%), with 64 providing diagnostic services and only 13 offering both core diagnosis and treatment. Prostate cancer facilities totalled 112 (16%), with 104 focused on diagnostic services and only 8 providing both. Figure 1a-e map facilities classified according to the core care criteria chosen by the expert panel over the population density per municipality. These maps show which facilities can provide core cancer care up to diagnostic suspicion compared to which facilities can provide a complete pathway to cancer diagnosis and treatment.

### Facilities providing enhanced cancer care

From the *N* = 14,133 facilities providing some type of care, and the *N* = 686 providing some type of cancer care, only *N* = 404 provided enhanced cancer care. Thus, even a smaller number of infrastructure is found to be enhanced. Breast, prostate and lung cancer care are the ones that least benefit from care provided in an enhanced environment. Figure 2a-e map the enhanced cancer care facilities per cancer type.

Although colon cancer shows high levels of availability of core facilities compared to other cancer types, it also has the highest difference in core and enhanced infrastructure, showing almost half of the facilities (43%) are not enhanced. In contrast, almost all lung cancer facilities that are core also classify as enhanced infrastructure, with only a 5% difference.

### Advanced infrastructure and human resources

**Fig. 3:**
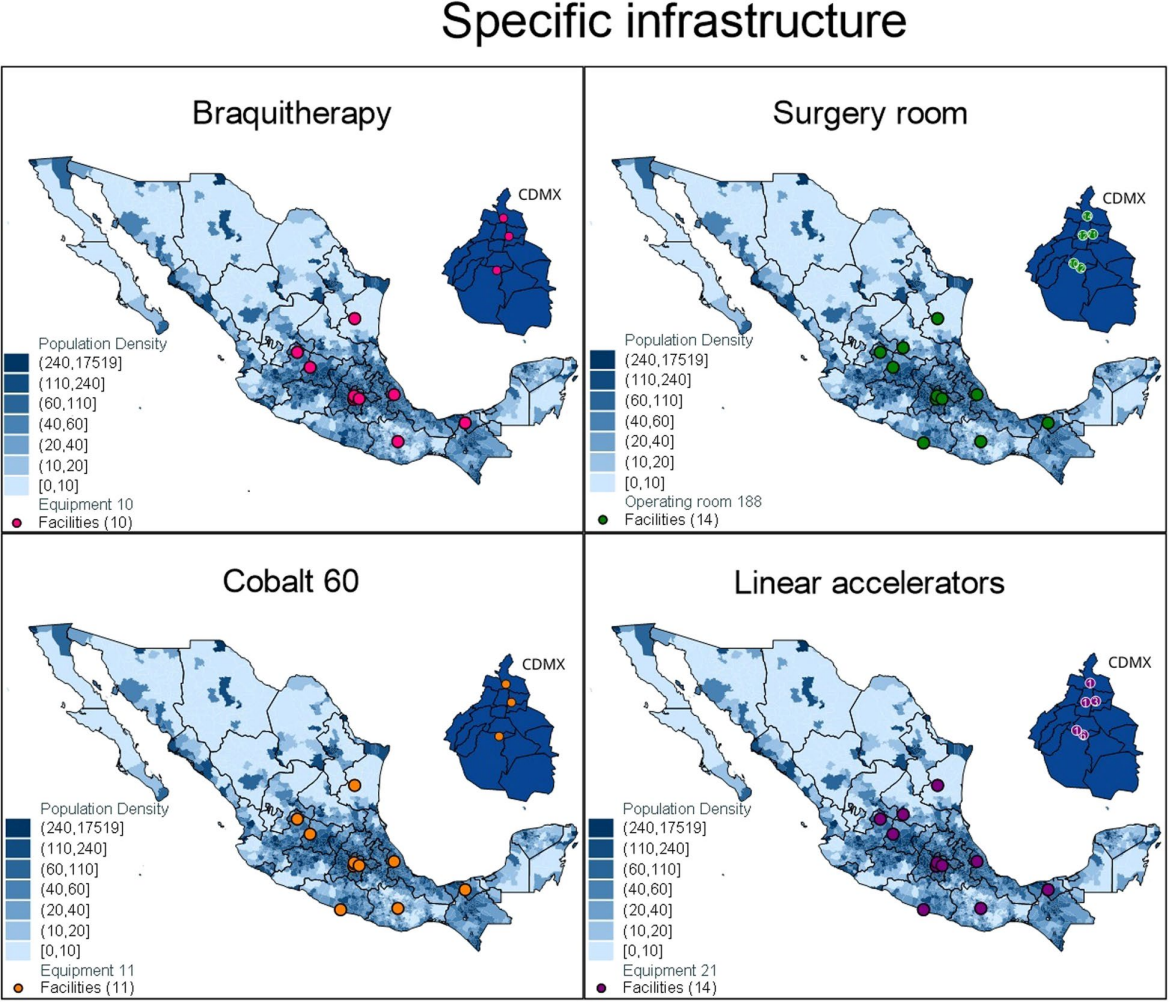
National distribution of brachytherapy, surgery, cobalt-60 and linear accelerators in Mexico

Cancer specific core or enhanced infrastructure that can provide diagnosis and radiotherapy or surgery at the national level included *N* = 10 brachytherapy units distributed in 10 facilities, *N* = 11 cobalt-60 in 11 facilities, *N* = 21 linear particle accelerators (LINAC) in 14 facilities, and *N* = 188 operating rooms distributed across 14 facilities. The distribution of therapeutic infrastructure for cancer diagnosis and treatment is visible in Fig. 3. Lastly, Table 4 shows the ratio of health oncologists per cancer care facility is 4.7.

**Table 4:** Ratio of health oncologists per cancer care facility identified

Category	Ratio (human resources/ non-cancer care facilities) (*N *= 14133)	Ratio (human resources/ cancer care facilities) (*N *= 686)
General Practitioners	3.8	23
Gynaecologists	6.8	7.9
Urologists	2.3	2.3
Pneumologists	2.8	2.8
Surgeons	5.9	6.1
Gastroenterologists	2.4	2.4
General Medical Oncologists	5	4.7
Specialist Nurses	12.2	25.4
General Nurses	9.5	92

## Discussion

Measuring the available infrastructure per state and/or by population density helps map available equipment and highlights disparities in infrastructure distribution. However, in our study, we decided to focus on mapping where patients can fulfil their pathway to diagnosis and treatment for each cancer type.

Our study not only provides an overview of the geolocation of the cancer care infrastructure offered by the Mexican public healthcare system for uninsured patients across the cancer continuum, but also sheds light on the potential challenges that patients, family members or caregivers face, such as transportation and unforeseen expenses, leading to lack or delayed access and worse outcomes [3, 7, 8, 45–51].

### Increase the number of facilities capable of undertaking full diagnosis and treatment

Mexico’s public health system needs a robust enhancement of the overall number of human resources and infrastructure to be able to respond to the country’s epidemiological needs [2, 8]. Our results demonstrate (1) lung, prostate and breast cancer require significant investments in diagnostic infrastructure compared to cervical and colon cancer and (2) the low availability of core therapeutic infrastructure across all types of cancer for people without social security coverage. Additionally, our results point to facilities currently providing core colon care are the ones in most need of enhancement to advance quality of life in these patients compared to the other cancer types.

Although the therapeutic infrastructure might seem higher than the facilities classified as being able to provide diagnosis and treatment, these are missing in the map due to not having the human resources or other infrastructure to be considered a facility that will be able to provide complete cancer care. For instance, the National Institute for Respiratory Diseases (INER) has cancer care specialists but no radiotherapy unit, therefore suggesting that patients diagnosed with lung cancer in this institution must navigate the health system to receive full treatment elsewhere. In some cases, just the LINAC is what prevents the facility from being visible in the maps. For instance, regional hospitals based in Guanajuato and Oaxaca lack a LINAC. Similarly, in a regional hospital in Tuxtla, Chiapas, LINAC, and other resources are lacking to be classified.

The mismatch between the human resources and the infrastructure explains why patients end up navigating the health system across states, because even if cancer is suspected or even diagnosed, they need to find where to access treatment as it is less accessible. As a result, both the public health system and the patient, the family or carer are enduring increased social and economic expenses and a complex cancer care journey [49, 52, 53].

### Regional and national decentralisation of available infrastructure

Previous literature [7, 54] has described resources to be centralised by identifying hospitalisations and specific institutions. Our maps show that while diagnosis infrastructure is not centralised, therapeutic resources are highly centralised particularly for lung, breast, and colon cancers. However, some states (i.e., Quintana Roo and Baja California Sur) do not have any centres available to provide any type of care for lung cancer. As a result, even if patients live close or in highly populated areas, they need to undertake very long journeys to reach for care. Which sometimes appears inaccessible to the most vulnerable population [49, 52, 53]. Due to centralisation, patient journeys may become discontinued from diagnostic suspicion to treatment, ultimately leading to delays in cancer care, late-stage diagnosis and worse survival outcomes [50, 55]. Our maps provide important information for state and municipal governments to understand care delivery gaps. Further research could be conducted to map the journeys taken by patients and the time it takes them to navigate the system.

Cities such as Mexico City, Guadalajara or Monterrey concentrate a larger number of specialists [2, 17, 19]. Thus, public policies must be formulated to decentralise healthcare services, ensuring equitable access to quality care across the territory [8]. Incentives must be identified to decentralise the labour force. This will not only reduce the burden on tertiary-level institutions in major cities but also make healthcare services more accessible to patients in remote areas.

### Equitable allocation of resources in densely populated areas

According to our maps, resources are not necessarily allocated in areas where population density seems higher. In a chain of events [56, 57], disparities in cancer resources allocation across municipalities might lead to inequalities in access to detection, early diagnosis, and treatment, as well as worsened outcomes and increased costs [58]. The unequal distribution of health infrastructure not only negatively affects health outcomes through the lack of access but also by the inability of different regions to train human resources [8]. Addressing these challenges requires a comprehensive approach that involves strengthening diagnostic capacities for cancer at the primary and secondary care levels [8, 59, 60]. More research should be done to understand the factors behind resource allocation, infrastructure, cancer incidence, and human development index to see if there is an association between the variables and the patient outcomes.

### Allocation of equitable infrastructure per cancer type

Our maps show there are enormous infrastructure differences by cancer types. This also supports the argument that analysing cancer as a single entity is inappropriate since all cancer types have a particular journey, infrastructure requirements, and barriers to care. National health policies to develop adequate and equitable infrastructure across cancer types is urgently needed [2]. More research needs to be done to calculate the effect of these infrastructure inequalities between cancer types in early diagnosis and treatment, survival, and quality of life.

Our resource classification shows that core infrastructure needs for prostate and lung cancer require more resources compared to other types of cancer. For instance, prostate cancer demands a comprehensive range of therapeutic infrastructure, including a gamma camera, radiotherapy services, and operating rooms [35–43]. Likewise, lung cancer demands a range of specialised resources such as pneumology, operating rooms, bronchoscopy, and radiotherapy [43]. In contrast, treatment for colorectal cancer primarily requires radiotherapy services and operating rooms [38]. Increased investment in currently unavailable core infrastructure in cancers such as prostate and lung cancer could help bridge the gap and promote more equitable health outcomes across cancer types.

### Obtaining precise and updated infrastructure data

Data is incomplete in the original datasets or not as detailed as suggested by the National Comprehensive Cancer Network Guidelines (NCCN) for resource stratification [44]. If we were to map the complete list of resources suggested by NCCN), the maps would only show a few numbers of facilities due to the missing data and thus misrepresent the availability of cancer resources in Mexico. Therefore, some assumptions needed to be made for this exercise, such as assuming institutions reporting oncologists can provide care to the different cancer types instead of relying on the full list of subspecialists. For example, oncological surgeons are not specified in the DGIS database, so we had to assume that hospitals with surgeons could provide basic surgical care to patients with cancer. Assumptions were also made for the codes such as “laboratory” (C1707) and “laboratory technician” (C2104), which were considered as available in the facility if either of them was marked as available. Additionally, radiotherapy in a database is not broken down into brachytherapy, cobalt60 or LINAC; hence although there were a small number of cases in this situation, this variable was coded as “available” interchangeably in either of the therapeutic resources. Another example of the impact of lack of precise data is paediatric hospitals identified as potential therapeutic infrastructure, when in fact we know for sure they do not provide care to adult cancers.

Clear and further specifications are needed to provide necessary information about cancer care, for example, the availability of a clinical and pathology laboratory to specify the types of exams that they can perform. The Supplementary Material 1 includes recommendations for the DGIS and CENETEC, for a more precise classification of infrastructure and resources. We labelled each variable as: (a) no changes needed, (b) disaggregation of the variable needed, (c) data needs to be collected again and included in the coding book, or (d) integration of codes necessary.

Precise and updated infrastructure data should be available to facilitate patient navigation in real time. In previous efforts. precise and real-time data on the facilities’ available infrastructure and functioning equipment facilitated the patient journey towards surgical procedures during the COVID-19 pandemic. Similar efforts should be placed in cancer infrastructure availability [61].

“Código Cáncer”, a project being conducted in Mexico, seeks to create a patient-transfer model in the public sector by referring patients to facilities that have the available infrastructure to reach diagnosis or treatment earlier in the disease continuum [61]. Collaborations between healthcare delivery institutions for the activation of local diagnostic centres equipped with imaging and testing facilities to enable prompt confirmation, accurate diagnosis, and rapid referral to facilities where treatment can be expedited is an essential component of any intervention. These sorts of collaborations might be an alternative for the overall shortage of diagnostic and treatment centres [25, 61]. However, innovative financing mechanisms should be put in place to be able to strive in such collaborations.

The creation of a real-time platform to inform on the availability of diagnostic infrastructure and treatment capacities in hospitals would potentially reduce delays in cancer care. Additionally, initiatives such as telemedicine [62–64] can be leveraged to bridge the gap and provide specialist consultations and support to primary care physicians in underserved municipalities. By decentralising healthcare services and implementing inclusive public policies, we can strive to provide quality care to all patients, irrespective of their insurance status [64]. This approach is likely to result in better patient survival rates and alleviate the financial burden that families often face in accessing healthcare services.

Our study has several limitations. Our maps rely on the data availability and hospitals reporting their resources, which sometimes may not be accurate. As an example, the databases show that *Instituto Nacional de Ciencias Médicas y Nutrición Salvador Zubirán*, one of the most equipped cancer centres in the country, does not have ultrasound, CT scan, radiotherapy unit, mammography equipment, or LINAC. In this case, we are certain that the DGIS database is inaccurate, and this might also be happening in other hospitals. More research needs to be done on the reasons why reporting these resources is not up to date or why it does not portray reality in some cases and what are the push and pull political or economic factors for underreporting.

The CENETEC database was last updated in 2016 and DGIS has not updated the database since 2022 [32, 33]. Thus, these maps might not account for changes in infrastructure that happened after. Moreover, the DGIS does not routinely collect data from the private hospitals regarding their infrastructure. Thus, these maps do not show the private health-care facilities that might provide care to patients. Therefore, it is currently not possible to map the areas with low infrastructure where the private sector might be more accessible. In any case, collecting data from the private sector routinely by the DGIS is encouraged, to be able to have resource data in hand in times of crises.

Another limitation from this study stems from the insufficient specificity of available information regarding whether the facilities meet the criteria and possess all the necessary resources for treating specific types of cancer. This issue is of paramount importance, given the common misconception that cancer is a singular disease when each cancer type necessitates highly specific elements for accurate diagnosis and treatment.

The existing public resources for assessing infrastructure suffer from a lack of uniformity and real-time updates. Additionally, variations in data reporting practices among different municipalities or regions and institutions may have influenced the results. However, this suggests that policies to ameliorate the quality of these databases are necessary. Finally, we assumed that reaching a facility with available infrastructure was equivalent to access to treatment, that all reported equipment is functional, and that human resources have available time slots to take care of patients. However, this may not be the case. Therefore, qualitative research should be done to collect data on the accessibility at the treatment stage in different centres to understand the barriers triggering the lack of resources throughout the year, using pre-defined DGIS codes.

## Conclusions

Our results provide relevant insight for allowing strategic resource allocation, paving the way for an enhanced healthcare landscape and improved health outcomes for the population.

Addressing disparities in cancer resources distribution in Mexico is a dual imperative—ensuring equity while seizing an opportunity to fortify the overall health system.

## Supplementary Information

Supplementary Material 1. Recommendations for further improvement in data collection.

Supplementary Material 2. Peer Review reports.

## Data Availability

Data is publicly available on official websites [32, 33].

## References

[1] Mohar-Betancourt A, Reynoso-Noverón N, Armas-Texta D, Gutiérrez-Delgado C, Torres-Domínguez JA. Cancer trends in Mexico: essential data for the creation and Follow-Up of public policies. J Glob Oncol. 2017;3(6):740–8. 29244991 10.1200/JGO.2016.007476 PMC5735971

[2] Brau-Figueroa H, Palafox-Parrilla A, Parrilla-Taylor P, Mohar A. Infraestructura oncológica En El sistema de Salud Mexicano. Salud Publica Mex. 2022;64(1):105–6. 35438910 10.21149/12739

[3] Gómez-Dantés O, Sesma S, Becerril VM, Knaul FM, Arreola H, Frenk J. Sistema de Salud de México. Salud Publica Mex. 2011;53(2):S220–32. 21877087

[4] Juan M, Moguel Ancheita A, Valdés Olmedo C, González Pier E, Martínez González G, Barraza Llorens M, Aguilera Aburto N, Trejo Rayón S, Soberón Acevedo G, Frenk Mora J, Ibarra Espinosa I, Lee GM, Tapia Conyer R, Kuri Morales P, Noriega Curtis C, Cano Valle F, Uribe Zúñiga P. Universalidad de los servicios de salud en México. Salud Publica Mex. 2013;55:1–64. 24570037

[5] Frenk J, Gómez-Dantés O. Health system in mexico. In: Levy A, Goring S, Gatsonis C, Sobolev B, van Ginneken E, Busse R, editors. Health services evaluation. New York: Springer US; 2019. p. 849–59.

[6] Gómez-Dantés O, Frenk J. Chronicle of a century of public health in Mexico: from public health to social protection in health. Salud Publica Mex. 2019;61(2):202–11. 30958963 10.21149/10122

[7] Gerson R, Zatarain-Barrón ZL, Blanco C, Arrieta O. Access to lung cancer therapy in the Mexican population: opportunities for reducing inequity within the health system. Salud Publica Mex. 2019;61(3):352–8. 31276352 10.21149/10118

[8] Goss PE, Lee BL, Badovinac-Crnjevic T, Strasser-Weippl K, Chavarri-Guerra Y, St Louis J, et al. Planning cancer control in Latin America and the Caribbean. Lancet Oncol. 2013;14(5):391–436. 23628188 10.1016/S1470-2045(13)70048-2

[9] Knaul FM, Arreola-Ornelas H, Touchton M, McDonald T, Blofield M, Avila Burgos L, et al. Setbacks in the quest for universal health coverage in Mexico: polarised politics, policy upheaval, and pandemic disruption. Lancet. 2023;402(10403):731–46. 37562419 10.1016/S0140-6736(23)00777-8

[10] Onofrei M, Vatamanu A-F, Vintilă G, Cigu E. Government health expenditure and public health outcomes: A comparative study among EU developing countries. Int J Environ Res Public Health. 2021;18(20):10725. 10.3390/ijerph182010725. 10.3390/ijerph182010725 PMC8535729 34682472

[11] Institute of Health Metrics and Evaluation (IHME). GBD Profile. Mexico. Institute for Health Metrics and Evaluation. Available from: http://www.healthmetricsandevaluation.org. [cited 2017 Jun 29].

[12] Organisation for Economic Co-operation and Development. OECD. Better policies for better lives. OECD. Available from: https://www.oecd.org/. [cited 2023 Aug 21].

[13] Global health research center at the University of Washington. IHME Measuring what matters. Institute of Health Metrics. Available from: http://www.healthdata.org/mexico. [cited 2019 Mar 19].

[14] OECD. OECD Reviews of Health Systems: Mexico 2016, OECD Reviews of Health Systems. Paris: OECD Publishing; 2016. 10.1787/9789264230491-en.

[15] OECD. OECD reviews of health systems: Mexico 2005. OECD; 2005.

[16] OECD Health Statistics. 2022 - OECD. Available from: https://www.oecd.org/els/health-systems/health-data.htm. [cited 2023 Jan 28].

[17] Heinze-Martin G, Olmedo-Canchola VH, Bazán-Miranda G, Bernard-Fuentes NA, Guízar-Sánchez DP. Los médicos especialistas En México. Gac Med Mex. 2018;154(3):342–51. 30047941 10.24875/GMM.18003770

[18] Trapani D, Murthy SS, Boniol M, Booth C, Simensen VC, Kasumba MK, et al. Distribution of the workforce involved in cancer care: a systematic review of the literature. ESMO Open. 2021;6(6):100292. 34763251 10.1016/j.esmoop.2021.100292 PMC8591344

[19] Flamand Gómez L, Moreno Jaimes C, Arriaga Carrasco R. Cáncer y desigualdades sociales en México, El Colegio de México. 2020. Available from: http://desigualdades.colmex.mx/cancer/informe-cancer-desigualdades-2020.pdf. [cited 2024 May 13]!ra eidción(978-607-564-239–0).

[20] de Santillana-Hernández SP, García-Flores MT, Galván-Oseguera H, Pérez-Rodríguez G, Martínez-Chapa HD. Diagnóstico situacional de la atención oncológica en el Instituto Mexicano del Seguro Social*. Revista Médica del Instituto Mexicano del Seguro Social; 2017. 29192747

[21] Biesecker C, Zahnd WE, Brandt HM, Adams SA, Eberth JM. A bivariate mapping tutorial for cancer control resource allocation decisions and interventions. Prev Chronic Dis. 2020;17:E01. 31895673 10.5888/pcd17.190254 PMC6977777

[22] Sudhof L, Amoroso C, Barebwanuwe P, Munyaneza F, Karamaga A, Zambotti G, et al. Local use of geographic information systems to improve data utilisation and health services: mapping caesarean section coverage in rural Rwanda. Trop Med Int Health. 2013;18(1):18–26. 23279379 10.1111/tmi.12016

[23] Yoon I, Twea P, Heung S, Mohan S, Mandalia N, Razzaq S, et al. Health sector resource mapping in Malawi: sharing the collection and use of budget data for Evidence-Based decision making. Glob Health Sci Pract. 2021;9(4):793–803. 34933976 10.9745/GHSP-D-21-00232 PMC8691869

[24] Gwede CK, Ward BG, Luque JS, Vadaparampil ST, Rivers D, Martinez-Tyson D, et al. Application of geographic information systems and asset mapping to facilitate identification of colorectal cancer screening resources. Online J Public Health Inf. 2010;2(1):2893. 10.5210/ojphi.v2i1.2893 PMC3615755 23569578

[25] Bautista-González E, Soto-Pérez-de-Celis E, Hasselkus-Sánchez GA, Muñoz Rocha TV, Gay-Molina JG, Ortiz-Blas LA et al. Código cáncer: resultados preliminares. GAMO; 2023.

[26] WHO IARC. Data factsheet Mexico, GLOBOCAN. Cancer Today. 2020. Available from: https://gco.iarc.fr/today/data/factsheets/populations/484-mexico-fact-sheets.pdf. [cited 2022 Apr 22].

[27] Arrieta O, Quintana-Carrillo RH, Ahumada-Curiel G, Corona-Cruz JF, Correa-Acevedo E, Zinser-Sierra J, et al. Medical care costs incurred by patients with smoking-related non-small cell lung cancer treated at the National Cancer Institute of Mexico. Tob Induc Dis. 2014;12(1):25. 25653577 10.1186/s12971-014-0025-4 PMC4316797

[28] Chávarri-Guerra Y, Villarreal-Garza C, Liedke PER, Knaul F, Mohar A, Finkelstein DM, et al. Breast cancer in Mexico: a growing challenge to health and the health system. Lancet Oncol. 2012;13(8):e335–43. 22846838 10.1016/S1470-2045(12)70246-2

[29] Barrios CH, Werutsky G, Mohar A, Ferrigno AS, Müller BG, Bychkovsky BL, et al. Cancer control in Latin America and the Caribbean: recent advances and opportunities to move forward. Lancet Oncol. 2021;22(11):e474–87. 34735817 10.1016/S1470-2045(21)00492-7

[30] Knaul FM, Arreola-Ornelas H, Velázquez E, Dorantes J, Méndez Ó, Ávila-Burgos L. El Costo de La Atención médica Del cáncer Mamario: El Caso Del Instituto Mexicano Del Seguro social. Salud Pública Méx. 2009;51:s286–95. 19967284 10.1590/s0036-36342009000800019

[31] Knaul FM, Arreola-Ornelas H, Wong R, Lugo-Palacios DG, Méndez-Carniado O. [The effect of Seguro popular de Salud on catastrophic and impoverishing expenditures in Mexico, 2004–2012]. Salud Publica Mex. 2018;60(2):130–40. 29738652 10.21149/9064

[32] Gobierno de México. Inventario Institucional de Datos de CENETEC. Datos abiertos. 2017. Available from: https://datos.gob.mx/busca/dataset/inventario-institucional-de-datos-de-cenetec. [cited 2024 Jun 23].

[33] Gobierno de México. Recursos en salud, nivel central. Datos abiertos. Available from: http://www.dgis.salud.gob.mx/descargas/datosabiertos/recursosSalud/Recursos_Salud_Sectorial_2022.zip?V=2023.11.10. [cited 2024 Jun 23].

[34] Pawson R, Tilley N. Realistic evaluation. 1st ed. SAGE Publications Ltd; 1997.

[35] Al Sukhun S, Temin S, Barrios CH, Antone NZ, Guerra YC, Chavez-MacGregor M, et al. Systemic treatment of patients with metastatic breast cancer: ASCO Resource-Stratified guideline. JCO Glob Oncol. 2024;10:e2300285. 38206277 10.1200/GO.23.00285 PMC10793992

[36] Shastri SS, Temin S, Almonte M, Basu P, Campos NG, Gravitt PE, et al. Secondary prevention of cervical cancer: ASCO Resource-Stratified guideline update. JCO Glob Oncol. 2022;8:e2200217. 36162041 10.1200/GO.22.00217 PMC9812449

[37] Chuang LT, Temin S, Berek JS. Management and care of patients with invasive cervical Cancer Resource-Stratified guideline expert panel. Management and care of patients with invasive cervical cancer: ASCO Resource-Stratified guideline rapid recommendation update. JCO Glob Oncol. 2022;8:e2200027. 35245079 10.1200/GO.22.00027 PMC8920468

[38] Chiorean EG, Nandakumar G, Fadelu T, Temin S, Alarcon-Rozas AE, Bejarano S, et al. Treatment of patients with Late-Stage colorectal cancer: ASCO Resource-Stratified guideline. JCO Glob Oncol. 2020;6:414–38. 32150483 10.1200/JGO.19.00367 PMC7124947

[39] Lopes G, Stern MC, Temin S, Sharara AI, Cervantes A, Costas-Chavarri A, et al. Early detection for colorectal cancer: ASCO Resource-Stratified guideline. J Glob Oncol. 2019;5:1–22. 30802159 10.1200/JGO.18.00213 PMC6426543

[40] Arrossi S, Temin S, Garland S, Eckert LO, Bhatla N, Castellsagué X, et al. Primary prevention of cervical cancer: American society of clinical oncology Resource-Stratified guideline. J Glob Oncol. 2017;3(5):611–34. 29094100 10.1200/JGO.2016.008151 PMC5646902

[41] Shaw SE, Paparini S, Murdoch J, Green J, Greenhalgh T, Hanckel B, et al. TRIPLE C reporting principles for case study evaluations of the role of context in complex interventions. BMC Med Res Methodol. 2023;23(1):115. 37179308 10.1186/s12874-023-01888-7 PMC10182844

[42] Litwin MS, Tan H-J. The diagnosis and treatment of prostate cancer: a review. JAMA. 2017;317(24):2532–42. 28655021 10.1001/jama.2017.7248

[43] National Institute for Health and Care excellence. Lung cancer: diagnosis and management. NICE Guidelines. 2019. Available from: https://www.nice.org.uk/guidance/ng122/chapter/Diagnosis-and-staging. [cited 2024 Mar 24]. 32119230

[44] Carlson RW, Scavone JL, Koh WJ, McClure JS, Greer BE, Kumar R, McMillian NR, Anderson BO. NCCN Framework for Resource Stratification: A Framework for Providing and Improving Global Quality Oncology Care. J Natl Compr Canc Netw. 2016;14(8):961–9. 10.6004/jnccn.2016.0103. 10.6004/jnccn.2016.0103 27496112

[45] Malalasekera A, Nahm S, Blinman PL, Kao SC, Dhillon HM, Vardy JL. How long is too long? A scoping review of health system delays in lung cancer. Eur Respir Rev. 2018;27(149):180045. 10.1183/16000617.0045-2018. 10.1183/16000617.0045-2018 PMC9488868 30158277

[46] Unger-Saldaña K. Challenges to the early diagnosis and treatment of breast cancer in developing countries. World J Clin Oncol. 2014;5(3):465–77. 25114860 10.5306/wjco.v5.i3.465 PMC4127616

[47] Unger-Saldaña K, Infante-Castañeda C. Delay of medical care for symptomatic breast cancer: a literature review. Salud Publica Mex. 2009;51(Suppl 2):s270–85. 19967283 10.1590/s0036-36342009000800018

[48] Unger-Saldaña K, Fitch-Picos K, Villarreal-Garza C. Breast cancer diagnostic delays among young Mexican women are associated with a lack of suspicion by health care providers at first presentation. J Glob Oncol. 2019;5:1–12. 31335236 10.1200/JGO.19.00093 PMC6690634

[49] Unger-Saldaña K, Ventosa-Santaulària D, Miranda A, Verduzco-Bustos G. Barriers and explanatory mechanisms of delays in the patient and diagnosis intervals of care for breast cancer in Mexico. Oncologist. 2018;23(4):440–53. 29284758 10.1634/theoncologist.2017-0431 PMC5896704

[50] Unger-Saldaña K, Miranda A, Zarco-Espinosa G, Mainero-Ratchelous F, Bargalló-Rocha E. Miguel Lázaro-León J. Health system delay and its effect on clinical stage of breast cancer: multicenter study. Cancer. 2015;121(13):2198–206. 25809536 10.1002/cncr.29331 PMC6681165

[51] Krok-Schoen JL, Brewer BM, Young GS, Weier RC, Tatum CM, DeGraffinreid CR, et al. Participants’ barriers to diagnostic resolution and factors associated with needing patient navigation. Cancer. 2015;121(16):2757–64. 25921981 10.1002/cncr.29414 PMC4529754

[52] Bautista-Gonzalez E, Hasselkus Sanchez GA. Barriers and facilitators to early lung cancer care in Mexico: A qualitative exploration from patients, relatives, and health professionals. Gaceta Mexicana de Oncología; 2024.

[53] Alcalde Castro M, Chavarri Guerra Y, Ramos-Lopez WA, Covarrubias-Gómez A, Sanchez S, Quiroz P, et al. Patient-reported barriers for accessing supportive care among patients with metastatic cancer treated at a public cancer center in Mexico. J Clin Oncol. 2018;36(34suppl):124–124.

[54] Hernández-Ávila JE, Palacio-Mejía LS, González-González L, Morales-Carmona E, Espín-Arellano LI, Fernández-Niño JA, et al. Utilization of hospital services for cancer care in Mexico. Salud Publica Mex. 2016;58(2):142–52. 27557372 10.21149/spm.v58i2.7783

[55] Bourlon MT, Remolina-Bonilla YA, Acosta-Medina AA, Saldivar-Oviedo BI, Perez-Silva A, Martinez-Ibarra N, et al. Impact of healthcare inequities on survival in Mexican patients with metastatic renal cell carcinoma. Front Oncol. 2023;13:1229016. 38044992 10.3389/fonc.2023.1229016 PMC10693405

[56] Kuh D, Ben-Shlomo Y. A life course approach to cancer epidemiology. In: Kuh D, Ben Shlomo Y, Ezra S, editors. A life course approach to chronic disease epidemiology. Oxford University Press; 2004. p. 260–80.

[57] Lynch J, Smith GD. A life course approach to chronic disease epidemiology. Annu Rev Public Health. 2005;26:1–35. 15760279 10.1146/annurev.publhealth.26.021304.144505

[58] Saunders CL, Abel GA, Lyratzopoulos G. Inequalities in reported cancer patient experience by socio-demographic characteristic and cancer site: evidence from respondents to the english Cancer patient experience survey. Eur J Cancer Care (Engl). 2015;24(1):85–98. 25327713 10.1111/ecc.12267 PMC4309492

[59] Biswas B, Talwar D, Meshram P, Julka PK, Mehta A, Somashekhar SP, et al. Navigating patient journey in early diagnosis of lung cancer in India. Lung India. 2023;40(1):48–58. 36695259 10.4103/lungindia.lungindia_144_22 PMC9894269

[60] Brouwers MC, Vukmirovic M, Tomasone JR, Grunfeld E, Urquhart R, O’Brien MA, et al. Documenting coordination of cancer care between primary care providers and oncology specialists in Canada. Can Fam Physician. 2016;62(10):e616–25. 27737997 PMC5063788

[61] Bautista-Gonzalez E, Aragón-Gama AC, Hasselkus Sanchez GA, Fernández YL. Major system change in surgeries during the Covid-19 pandemic: reflections on rapid service adaptation among private and public institutions in Mexico. SSRN J. 2023. 10.2139/ssrn.4449332.

[62] Coelho KR. Identifying telemedicine services to improve access to specialty care for the underserved in the San Francisco safety net. Int J Telemed Appl. 2011;2011:523161. 22187550 10.1155/2011/523161 PMC3236479

[63] Salman OH, Aal-Nouman MI, Taha ZK. Reducing waiting time for remote patients in telemedicine with considering treated patients in emergency department based on body sensors technologies and hybrid computational algorithms: toward scalable and efficient real time healthcare monitoring system. J Biomed Inf. 2020;112:103592. 10.1016/j.jbi.2020.103592 33091572

[64] Shah TK, Tariq T, Phillips R, Davison S, Hoare A, Hasan SS, et al. Health care for all: effective, community supported, healthcare with innovative use of telemedicine technology. J Pharm Policy Pract. 2018;11:3. 29435335 10.1186/s40545-018-0130-5 PMC5793345

