# Supplementary material for: Assessing disparities in cancer resources distribution in Mexico
Source: BMC Health Serv Res. 2025 Apr 17;25:564. doi: 10.1186/s12913-025-12497-z (PMC12007217; doi:10.1186/s12913-025-12497-z)
Supplement: Supplementary file 1 — Supplementary Material 1. Recommendations for further improvement in data collection. [file 12913_2025_12497_MOESM1_ESM.docx]

**Additional File A:** Recommendations for further improvement in data collection

| **Source** |  | **DGIS CODE** | **Status** | **Justification** |
| --- | --- | --- | --- | --- |
|  | **Human resources for health** | | | |
| DGIS | General or Primary care Practitioner or Physician | C1301 | No changes needed | — |
|  | General Nurse | C2101 | No changes needed | — |
|  | Specialised nurses | C2102 | No changes needed |  |
|  | Obstetrician-gynaecologist | C1804 | No changes needed | — |
|  | Surgeon (general and/or specialised) | C1805 | Disaggregation is needed | Different surgical specialties are involved in various types of cancers’ care, including cardiothoracic surgeon, oncology surgeons: onco-gynaecology surgeon, colorectal surgeon, or oncology-gynaecologist. These can replace C1804, C1816, C1819, |
|  | Gastroenterologist | C1816 | Disaggregation is needed | — |
|  | Urologist | C1819 | No changes needed | — |
|  | Plastic and reconstructive surgeon | C1820 | No changes needed | — |
|  | Pneumologist | C1821 | No changes needed | — |
|  | Oncologist (non-surgeon) | C1823 | Disaggregation is needed | Needed to specify if an oncologist is either a medical or radiation specialist. |
|  | Anatopathologist | C2004 | Integration of codes | — |
|  |  | C2412 | (It would be useful to specify the differences between these codes: histopathologist, cytotechnologist, and specialist in anatomopathology or integrate them) |  |
|  |  | C2413 |  |  |
|  | Laboratory technician | C2404 | No changes needed | — |
|  |  |  | (Although no changes are necessary, it would be useful to specify the capacities found in each technician) |  |
|  | Radiology technician | C2409 | No changes needed | — |
|  |  |  | (Although no changes are necessary, it would be useful to specify the capacities found in each technician) |  |
| Not included | Endoscopist | Not collected nor coded | Data needs to be collected from scratch and included in the coding book | Necessary for specific cancer types. In Mexico, various medical specialists can take specific endoscopic training. |
|  | Radiation-oncology | Not collected nor coded | Data needs to be collected from scratch and included in the coding book. | Needed to operate and prescribe cancer radiation therapy |
|  | Palliative care specialist | Not collected nor coded | Data needs to be collected from scratch and included in the coding book | Needed for end-of-life treatment and patient relief |
|  | Radiologist | Not collected nor coded | Data needs to be collected from scratch and included in the coding book | Needed for interpretation of specific cancer types and complex cases. Needed to specify differences with a radiology technician. |
|  | Medical geneticist | Not collected nor coded | Data needs to be collected from scratch and included in the coding book | Needed for genetic counselling for hereditary risk of cancer. |
| **Diagnosis infrastructure** | | | | |
|  | Interventional radiology | Not collected nor coded | Data needs to be collected from scratch and included in the coding book | Needed to perform complex biopsies and specific targeted therapies. |
| DGIS | Clinical Laboratory | C1707 | Disaggregation is needed | — |
|  |  |  | (Specify the studies that are capable of performing, i.e. Prostatic Specific Antigen, Carcinoembryonic antigen, hormonal receptors determination, HER, EGFR / ALK / ROS1 mutation, RAS and BRAF Mutations ) |  |
|  | Ultrasound | C1710 | No changes needed | — |
|  | Tomography | C1735 | No changes needed | — |
|  | Mammography | C1747 | No changes needed | — |
|  | Magnetic Resonance Imaging | C1749 | No changes needed | — |
| EMAT 2016 | PET/CT | Not coded | Data needs to be collected from by DGIS and included in the coding book | Needed to assess specific and variable cancer types, before or during treatment. Radiotracers vary between cancer types (FDG, PSMA). |
| Not included | Endoscopy equipment | Not collected nor coded | Data needs to be collected from scratch and included in the coding book | Needed for diagnosis and treatment of complications of certain cancer types, usually gastrointestinal ones. It is assumed that there is a specific room to perform the procedure. |
|  | Microsatellite instability determination | Not collected nor coded | Data needs to be collected from scratch and included in the coding book | Needed to assess genomic molecular stability, predicting response to immunotherapy or presence/absence of Lynch syndrome. |
|  | Anatomy Pathology laboratory | Not collected nor coded | Data needs to be collected from scratch and included in the coding book | Needed to specify diagnosis, staging and/or molecular analysis |
|  | Cystoscopy | Not collected nor coded | Data needs to be collected from scratch and included in the coding book | Needed to assess, diagnose and treat malignancies or other diseases in the bladder and prostate. |
|  | Bronchoscopy | Not collected nor coded | Data needs to be collected from scratch and included in the coding book | Needed to assess, diagnose and treat malignancies or other diseases in the trachea, bronchus and lungs. It is assumed that there is a specific room to perform the procedure. |
|  | Respiratory function tests or spirometry | Not collected nor coded | Data needs to be collected from scratch and included in the coding book | Needed to assess the physiology or function of the lung, usually before or during treatment for lung cancer or other thoracic malignancies. |
| **Therapeutic infrastructure** | | | | |
| DGIS | Surgical rooms | C1701 | Disaggregation is needed | Needed to specify the type of surgeries and equipment that can be performed in them. I.e. urology surgeries, cardiothoracic surgeries, gynaecology surgeries etc. |
| DGIS | Radiotherapy unit | C1738 | No changes needed | No changes needed |
|  | Linear accelerator | C1730 | No changes needed | No changes needed |
| EMAT 2016 | Gamma-camera, gamma-scan or gamma-graph | Not coded | Data needs to be collected from scratch and included in the coding book | Needed to diagnose extension of specific cancer types, usually bone metastases of either lung, breast or prostate cancer. |
|  | Automatic brachytherapy | Not coded | Needed to treat specific cancer types | Needed to treat specific cancer types |
|  | Cobalt 60 Pump | Not coded | Needed to treat specific cancer types | Needed to treat specific cancer types |
| Not included | Chemotherapy mixing or preparation unit | Not collected nor coded | Data needs to be collected from scratch and included in the coding book | Needed for safe preparation of chemotherapy |
|  | Chemotherapy application unit | Not collected nor coded | Data needs to be collected from scratch and included in the coding book | Needed for safe administration of chemotherapy |
