# Supplementary material for: Assessing disparities in cancer resources distribution in Mexico
Source: BMC Health Serv Res. 2025 Apr 17;25:564. doi: 10.1186/s12913-025-12497-z (PMC12007217; doi:10.1186/s12913-025-12497-z)
Supplement: Supplementary file 2 — Supplementary Material 2. Peer Review reports. [file 12913_2025_12497_MOESM2_ESM.pdf]

	COMMENT	ORIGINAL	AUTHORS COMMENTS	MODIFICATION STATUS	NEW VERSION
REVIEWER 1	Given the specific topic and the focus of the study, it would be pertinent to indicate which Sustainable Development Goals (SDGs) the study aligns with.	This study seeks to understand existing gaps in cancer resource distribution in Mexico, focusing on health facilities for the uninsured population with the goal of informing policy and advocating for the enhancement of healthcare infrastructure in the Ministry of Health.	We have added the correspondant SDG.	Changed	This study seeks to understand existing gaps in cancer resource distribution in Mexico, particularly in health facilities serving the uninsured population, with the goal of informing policy and advocating for healthcare infrastructure improvements within the Ministry of Health. In alignment with the United Nations Sustainable Development Goals (SDGs), the project supports SDG 3 (Good Health and Well-being) by promoting equitable access to cancer care, SDG 10 (Reduced Inequalities) by addressing regional and population disparities, and SDG 9 (Industry, Innovation, and Infrastructure) by advocating for the development of resilient and accessible health infrastructure.
	The databases analyzed (second paragraph of the Methods section), are they publicly available? Please specify, and if so, provide the URL.	Relevant datasets were selected based on their potential to provide comprehensive information on oncological infrastructure, including facilities, equipment, and services for the public healthcare facilities for non-insured patients. These were accessible through official health-related websites in Mexico i.e. from the General Direction of Health Information (DGIS), Health Sectorial Resources 2022 and from the National Centre of Excellence Technology in Health (CENETEC-Salud), the High Specialty Medical Equipment National Inventory (EMAT) 2016 (29,30).	We have specified that these datasets were publicly available. However, the URL was already in the references and was not added in the original text to avoid increasing the word count. We have made sure the URL was still accesible.	Partially changed	Publicly available datasets were sought and selected based on their potential to provide comprehensive information on oncological infrastructure, including facilities, equipment, and services for the public healthcare facilities for non-insured patients. These were accessible through official health-related websites in Mexico i.e. from the General Direction of Health Information (DGIS), Health Sectorial Resources 2022 and from the National Centre of Excellence Technology in Health (CENETEC-Salud), the High Specialty Medical Equipment National Inventory (EMAT) 2016 (34,35).
	In the third paragraph of the Methods section, it is stated that duplicate facilities were removed. How many were there? Please include this information in the manuscript, as it is highly relevant. In Mexico, and for multiple reasons beyond the scope of this manuscript, official records may identify medical units that ceased to function years ago.	Facilities in Mexico were identified through a unique identifying number (CLUES) according to official records at DGIS databases. Datasets were merged and duplicate facilities were deleted.	We have made the merging process clear and decisions were made over the variables and datasets selected.	Changed	Facilities in Mexico were identified through a unique identifying number (CLUES). Datasets were merged and in the case of duplicaton of resources per facility the most recent resources were considered for the analysis. The source of each resource considered is available in the first column of Table 1.
	Please consider making the triangles in the symbols for Figures 1a-e smaller. They overlap and make visualization difficult.		Thank you for the observation. We have made the icons smaller.	Changed	Changed
	The authors found that infrastructure investment in health has been heterogeneous among the cancers analyzed, and cervical and colon cancers have not been favored. Despite this, and largely due to the specific protection provided by the vaccine that has been administered for over a decade, the burden associated with cervical cancer has decreased. I suggest mentioning this briefly in the discussion section.	Our resource classification shows that the infrastructure needs for prostate and lung cancer require more public health investment compared to other types of cancer. Prostate cancer demands a comprehensive range of therapeutic infrastructure, including a gamma camera, radiotherapy services, and operating rooms. Likewise, lung cancer demands a range of specialised resources such as pneumology, operating rooms, bronchoscopy, and radiotherapy. In contrast, treatment for colorectal cancer primarily requires radiotherapy services and operating rooms. Increased investment in currently unavailable specialised infrastructure for high-incidence cancers such as prostate and lung cancer could help bridge the gap and promote more equitable health outcomes across these types of cancer.	Cervical cancer burden of disease in Mexico has not decreased despite vaccination. **Cruz-Valdez A, Palacio-Mejía LS, Quezada-Sánchez AD, Hernández-Ávila JE, Galicia-Carmona T, Cetina-Pérez LDC, Arango-Bravo EA, Isla-Ortiz D, Aranda-Flores CE, Uscanga-Sánchez SR, Madrid-Marina V, Torres-Poveda K. Cervical cancer prevention program in Mexico disrupted due to COVID-19 pandemic: Challenges and opportunities. Front Oncol. 2023 Mar 9;13: 1008560. doi: 10.3389/fonc.2023.1008560. PMID: 36969022; PMCID: PMC10034019]**	Not changed	Not changed
R 2	1) Highlighting other factors that contribute to GDP would be helpful. Ex: Investment, government spending, consumption, Labor force, Political stability, inflation, etc. 2) It might be helpful for readability that authors establish a connection between GDP and health care infrastructure, despite less quality and accessibility.	In 2022, Mexico allocated 5.5% of its GDP to healthcare, which is below the Organisation for Economic Co-operation and Development (OECD) average(8). The number of hospital beds per 1000 inhabitants in Mexico is also significantly lower than the OECD average of 4.8, standing at a mere 1.5 (8–12).	We have added this concept to the paragraph	Changed	Although macro economic factors influence the proportion of the GDP allocated to healthcare, they can sometimes restrict the development of healthcare infrastructure and the recruitment of healthcare personnel (new ref) . In 2022, Mexico allocated 5.5% of its GDP to healthcare, which is below the Organisation for Economic Co-operation and Development (OECD) average(8). This limited allocation is reflected in the country's healthcare infrastructure, where the number of hospital beds per 1000 inhabitants in Mexico is also significantly lower than the OECD average of 4.8, standing at a mere 1.5.
	The first paragraph is vague for international readers. The authors need to provide more background on how the healthcare system works in Mexico. For example, is Cancer screening provided for free? Is basic treatment covered? Etc. Not very detail but some background would be helpful.	Mexican healthcare system suffers from the paradox of over-centralization, as well as system fragmentation, as well as lack of universal health coverage. There are three main health care providers, those that provide private care, public social security institutions that provide care to the formally employed and the ministry of health that provides care to the otherwise uninsured, unemployed or informally employed.	We have integrated the recommendations into the paragraph.	Changed	In Mexico there are three main health care providers, those that provide private care, public social security institutions that provide care to the formally employed and the ministry of health that provides care to the otherwise uninsured, unemployed or informally employed (2,4,5). Each one of these providers has the governance over what diseases are covered and how resources are allocated and ultimately distributed (2,5–7). Moreover, the Mexican healthcare system suffers from the paradox of over-centralization, fragmentation and lack universal health coverage(1–3), resulting in. inaccesible cancer screening, diagnosis or treatment according to cancer type and affiliation (1–7).
	Finding oncologists in rural areas is a common problem across the board. Recommend authors to explore if there are programs in Mexico that help promote specialists to practice in rural areas. https://www.ncbi.nlm.nih.gov/books/NBK316259/	Specifically for cancer care, the number of specialists is well below the internationally recommended level and insufficient to meet the country's healthcare needs(10). One study conducted in 2018 measured the number of specialists per state and found there were 0.71 oncology surgeons, 0.6 paediatric oncology surgeons, 0.31 oncologists, and 0.29 gynaecologist-oncologists per 1000 habitants, all concentrated in urban areas, particularly in the three largest cities in Mexico(10). Moreover, studies looking at cancer related infrastructure have also found significant deficits, as well as concentrations in privileged urban settings, in close proximity to high socio-economic populations (11)(12)(13). However, the public healthcare system covering the uninsured population has not mapped the availability of resources needed according to population density nor per cancer type needs.	The article suggested by reviewer indeed discusses programs aimed at increasing the presence of general healthcare providers in rural areas, such as the Social Service Year and Coplamar, which focused on primary care. These programs did not explicitly target specialists like oncologists. However, the reviewer raises an important point about the need for specialized care in rural regions, particularly for diseases like cancer.	Not changed	
	The last sentence about "geographical assessment of the human resources and infrastructure…" is inconsistent with the abstract. I recommend correcting one of them and making things consistent.	Geolocalisation of healthcare resources has proven to facilitate policy-making for health resource allocation (14–17). Hence, a patient-centred geographical assessment of the human resources and infrastructure allocated for each cancer type is necessary for healthcare planning and achieving an equitable distribution of resources(7). This study seeks to understand existing gaps in cancer resource distribution in Mexico, focusing on health facilities for the uninsured population with the goal of informing policy and advocating for the enhancement of healthcare infrastructure in the Ministry of Health.	Thank you for the observation, we have changed the sentence so it is in accordance with our findings and sentence building.	Changed	Mapping healthcare resources (or the lack off) has proven to facilitate policy-making for more equitable(6)resource allocation in other contexts (19–22). However, the public healthcare system covering the uninsured population in Mexico has simply quantified each individual resource (i.e. number of CT scan per region, number of specialists) but has not geographically mapped resources considering all the patient needs across the cancer continuum or journey(i.e. laboratory, CT scan, nurses, oncologists, pathologist, nutritionist, etc) nor has it cross-mapped resources with population density .

REVIEWE	Under the methods section, authors could develop a table that lists data variables and their corresponding data sources.	As part of the project(18), this analysis employed a cross-sectional study design to assess the available diagnostic and therapeutic infrastructure for five most relevant cancer types in Mexico: breast, lung, prostate, colorectal and cervix (18). These site specific cancers were chosen due to high incidence-mortality-ratios and impact as catastrophic expenditures (7,19–21). A realist framework informed the analysis, drawing on existing literature on cancer infrastructure assessment (22–28). Relevant datasets were selected based on their potential to provide comprehensive information on oncological infrastructure, including facilities, equipment, and services for the public healthcare facilities for non-insured patients. These were accessible through official health-related websites in Mexico i.e. from the General Direction of Health Information (DGIS), Health Sectorial Resources 2022 and from the National Centre of Excellence Technology in Health (CENETEC-Salud), the High Specialty Medical Equipment National Inventory (EMAT) 2016 (29,30). **Facilities in Mexico were identified through a unique identifying number (CLUES) according to official records at DGIS databases. Datasets were merged and duplicate facilities were deleted.** To assess regional disparities, the information extracted from the database categories was mapped to identify which municipalities held available infrastructure and was cross mapped with municipal population density to provide visual aid on the geographical distribution of facilities by population.<br>The development of the categorization scheme for the infrastructure and human resources for cancer was based on the National Comprehensive Cancer Network guidelines. However, to ensure the accuracy, appropriateness for the Mexican context, and consider the available data, systematic validation process was implemented, involving pilot testing and cross validation with expert panel, consulting with six domain experts in oncology and public health in Mexico. As a result, two infrastructure categories arose from expert consensus: facilities that can suspect cancer and provide a preliminary diagnosis and facilities that confirm diagnosis and stage, identifies cancer subtypes, and provides treatment.<br>Additionally, the designation "INDISP" was used to classify resources indispensable for a core diagnosis and treatment of a patient. Alternatively, the "EXPEND" designation represents elements that, following a patient-centred logic, would be required for enhanced care of the patient; however, their availability did not hinder management or impair diagnosis and treatment. Table 1 describes the infrastructure typology developed and Table 2 shows the resources necessary for preliminary diagnosis or diagnosis and treatment and the resources used to classify health facilities into core or enhanced.<br>To avoid over-exclusion, we did not consider general practitioners as an exclusion criterion for facilities in the complete pathway category. It was assumed that in most specialised hospitals, which focus on specific diseases like cancer, would not typically employ general practitioners, instead, these hospitals would rely on	The variables and sources are now available in Table 1. This shows the variable codes of each database and the resources needed to meet the classifications proposed.	Changed	Facilities in Mexico were identified through a unique identifying number (CLUES). Datasets were merged and in the case of duplicaton of resources the most recent resources were considered for the analysis. The source of each resource considered is available in the first column of Table 1.
	We need more clarity on how the data was merged. Did the authors identify all the information specific to each cancer type?		Yes, first we identified the resources necessary for each cancer type and then we classified the facilities according to the availability of the data. We solved this concern further specifying the merger and what dataset overtook the other. We did not weigh the databases, we decided to use the data that was most recently available. In addition, we have edited the methods section to ensure its comprehension. We changed some of the original text and made sure to include more detail.	Changed	Study design<br>As part of the BLINDED roject(23), five site specific cancers were chosen for this cross sectional study, due to high incidence-mortality-ratios and impact as catastrophic expenditures (6,24–26). This analysis identified the available diagnostic and therapeutic infrastructure for five major cancer types in Mexico: breast, lung, prostate, colorectal, and cervical cancers (23).<br>Data source and eligibility<br>Publicly available datasets were sought and selected based on their potential to provide comprehensive information on oncological infrastructure, including facilities, equipment, and services for the public healthcare facilities for non-insured patients. These were accessible through official health-related websites in Mexico i.e. from the General Direction of Health Information (DGIS), Health Sectorial Resources 2022 and from the National Centre of Excellence Technology in Health (CENETEC-Salud), the High Specialty Medical Equipment National Inventory (EMAT) 2016 (27,28). Facilities in Mexico were identified through a unique identifying number (CLUES). Datasets were merged and in the case of duplicaton of resources the most recent resources were considered for the analysis. The source of each resource considered is available in the first column of Table 1.<br>Data analysis Six domain experts in oncology and public health in Mexico were involved in initially identifying the diagnostic and therapeutic resources needed by each cancer type. Infrastructure and human resources were categorized and selected per cancer based on existing ASCO Guidelines (30–36) and on the National Comprehensive Cancer Network Guidelines for resource stratification (37). However, to ensure that these were categorised accurately for the Mexican context, consensus was made between the experts to develop a classification based on the available data. As a result, from the merged resources database, two resource categories arose from consensus: service scope (diagnostic or diagnostic and therapeutic) and infrastructure level (core or enhanced). Table 1 shows the resources available from each dataset and how each one was classified in both service scope and infrastructure level for each cancer type. If deemed unnecesary for the cancer type in question, the cell was coloured in gray. The designation "INDISP" was used to classify each resource when they were indispensable for core patient care and alternatively, the "EXPEND" designation was used when, resources would enhance patient care. The rationale for this "EXPEND" was to include facilities that may provide enhanced cancer care through physical therapy, nutritionists or other infrastructure alternatives in addition to diagnosis or treatment.
	Lot of inconsistencies with the methodology. A diagram (decision tree) depicting how resource availability was coded and if there was a weightage consideration for density would be helpful.				
	Overall, sentence framing could be bolstered to help increase readability.	Full text	Thank you for the observation. We have tried to make the sentences very clear.	Changed	See full text.
	References are out of order.		Thank you for the observation	Changed	References are now in order. Thank you for the observation.
	Unclear what the author is trying to state with this sentence: "Geolocalisation of healthcare resources has proven to facilitate policymaking for health resource allocation."	Geolocalisation of healthcare resources has proven to facilitate policy-making for health resource allocation (14–17).	In this paragraph, we are discussing the importance of using geolocalization (geographic data and mapping) to improve decision-making when allocating healthcare resources, specifically for cancer treatment. Geolocalization of healthcare resources: This refers to mapping where healthcare facilities, medical professionals, and equipment are located. It helps policymakers visualize the distribution of these resources across different regions, making it easier to identify where there are shortages or gaps.	Changed	Mapping healthcare resources (or the lack off) has proven to facilitate policy-making for more equitable(6)resource allocation in other contexts (19–22).
	Check references order	References	Thank you for the observation	Changed	References are now in order. Thank you for the observation.
	Paragraph 2. Authors are comparing Mexico with Finland, I don't think it's a valid comparison due to so many differences: country income, population, health system. It would be more useful to compare with similar countries.	Mexico lags OECD benchmarks in healthcare personnel, with only 2.5 doctors and 2.9 nurses per 1000 inhabitants (13). These figures are similar to those of other middle-income countries such as Turkey and Brazil, yet they fall short when compared to high-income countries(13). For example, Finland, a high-income country, boasts nearly seven times more healthcare professionals per capita. This stark contrast underscores the significant healthcare workforce capacity gap between Mexico and high-income nations.	We have first compared it to Turkey and Brazil, and then to Finland as part of the OECD (the higuest score).	Changed	Additionally, Mexico lags OECD benchmarks in healthcare personnel, with only 2.5 doctors and 2.9 nurses per 1000 inhabitants (13). These figures are similar to those of other middle-income countries in the OECD such as Turkey and Brazil, yet they fall short when compared to high-income countries like Finland with nearly seven times more healthcare professionals per capita(13). This contrast underscores the significant healthcare workforce capacity gap between Mexico and high-income nations.
	Paragraph 3, sentence 1. Please state the recommended level of specialists to give the reader something to compare with. Also, an official internationally recognized reference is needed to support this.	Specifically for cancer care, the number of specialists is well below the internationally recommended level and insufficient to meet the country's healthcare needs(13). One study conducted in 2018 measured the number of specialists per state and found there were 0.71 oncology surgeons, 0.6 paediatric oncology surgeons, 0.31 oncologists, and 0.29 gynaecologist-oncologists per 1000 habitants, all concentrated in urban areas, particularly in the three largest cities in Mexico(13).	We have added the required data from an internationally recognized reference.	Changed	Specifically for cancer care, the number of specialists is well below the internationally recommended level and insufficient to meet the country's healthcare needs, which is 20 full-time hired oncologists per million of habitants (13, [NEW REF]). One study conducted in 2018 measured the number of specialists per state and found there were 0.07 oncology surgeons, 0.06 paediatric oncology surgeons, 0.03 oncologists, and 0.02 gynaecologist-oncologists, a total of 0.18 non-specific oncologists per million of habitants, all concentrated in urban areas, particularly in the three largest cities in Mexico (12).
	Paragraph 3, last sentence: not clear what you mean. If this is the research gap, it needs to be stated more clearly.	However, the public healthcare system covering the uninsured population has not geographically mapped the availability of resources needed according to population density nor per cancer type needs.	We have modified paragraph so the research gap and objective of the paper is clear. We changed some of the original text and made sure to include more detail.	Changed	However, the public healthcare system covering the uninsured population in Mexico has simply quantified each individual resources (i.e. number of CT scan per region, number of specialists) but not geographically mapped the availability of resources taking into account all the resources needed across the full cancer continuum (i.e. laboratory, CT scan, nurses, oncologists, pathologist, nutritionist, etc) nor has it cross-mapped resources with population density. This study seeks to understand existing gaps in cancer resource distribution in Mexico, particularly in health facilities serving the uninsured population, with the goal of informing policy and advocating for healthcare infrastructure improvements within the Ministry of Health.

	Paragraph 4, sentence 1. What is geolocalisation of healthcare resources? What is the evidence behind the statement “has proven to facilitate policy-making for health resources allocation”? And what does “facilitate” mean?	Geolocalisation of healthcare resources has proven to facilitate policy-making for health resource allocation (14–17). Hence, a patient-centred geographical assessment of the human resources and infrastructure allocated for each cancer type is necessary for healthcare planning and achieving an equitable distribution of resources(7). This study seeks to understand existing gaps in cancer resource distribution in Mexico, focusing on health facilities for the uninsured population with the goal of informing policy and advocating for the enhancement of healthcare infrastructure in the Ministry of Health.	Geolocalization refers to mapping the geographical location of healthcare resources, such as facilities and personnel. The evidence supporting this approach comes from studies referenced, demonstrating that mapping helps policymakers make more equitable decisions by showing where healthcare gaps exist. In this context, "facilitate" means making the process of allocating resources more efficient and effective by visually identifying disparities. However, in Mexico’s public healthcare system, resource allocation has been based on quantifying individual resources without considering full cancer care needs or population density. We will clarify this distinction in the revised text.<br><br>Thank you for the suggestion.	Changed	Mapping healthcare resources (or the lack off) has proven to facilitate policy-making for more equitable(6)resource allocation in other contexts (19–22). However, the public healthcare system covering the uninsured population in Mexico has simply quantified each individual resource (i.e. number of CT scan per region, number of specialists) but has not geographically mapped resources considering all the patient needs across the cancer continuum or journey(i.e. laboratory, CT scan, nurses, oncologists, pathologist, nutritionist, etc) nor has it cross-mapped resources with population density .
	Paragraph 4, sentence 2. Not clear to me the argument following from sentence 1 to 2, as to use the “hence”.<br>o Do the authors mean “recommended”? By the Latin American Cancer Lancet Commission they are referring to?<br>o	Specifically for cancer care, the number of specialists is well below the internationally recommended level and insufficient to meet the country’s healthcare needs(13). One study conducted in 2018 measured the number of specialists per state and found there were 0.71 oncology surgeons, 0.6 paediatric oncology surgeons, 0.31 oncologists, and 0.29 gynaecologist-oncologists per 1000 habitants, all concentrated in urban areas, particularly in the three largest cities in Mexico(13).	A newer reference, a full global analysis from ESMO on Workforce on not only oncologists but full-time employed oncologists was used, found to be more thorough than the Latin American Lancet Comission held by 2015. Original data was corrected.	Changed	Specifically for cancer care, the number of specialists is well below the internationally recommended level and insufficient to meet the country’s healthcare needs, which is 20 full-time hired oncologists per million of habitants (13, [NEW REF]). One study conducted in 2018 measured the number of specialists per state and found there were 0.07 oncology surgeons, 0.06 paediatric oncology surgeons, 0.03 oncologists, and 0.02 gynaecologist-oncologists, a total of 0.18 non-specific oncologists per million of habitants, all concentrated in urban areas, particularly in the three largest cities in Mexico (12).
	Paragraph 4, sentence 2. Not clear to me the argument following from sentence 1 to 2, as to use the “hence”.<br>o What is a “patient-centered geographical assessment”?<br>o Why is a “patient-centered geographical assessment of human resources and infrastructure” allocated for each cancer “necessary”?<br>o How do you know that it wasn’t planned in this way by Mexican policy makers? Maybe it would be more useful to phrase differently, stating that this study intends to assess existing geogrpahical distribution resources in comparison with…	Geolocalisation of healthcare resources has proven to facilitate policy-making for health resource allocation (14–17). Hence, a patient-centred geographical assessment of the human resources and infrastructure allocated for each cancer type is necessary for healthcare planning and achieving an equitable distribution of resources (7). This study seeks to understand existing gaps in cancer resource distribution in Mexico, focusing on health facilities for the uninsured population with the goal of informing policy and advocating for the enhancement of healthcare infrastructure in the Ministry of Health.	We have deleted this from the manuscript to avoid confusion and rephrased in the justification of the project what our aim was. We have also provided more examples to make it more clear.	Changed	Mapping healthcare resources (or the lack off) has proven to facilitate policy-making for more equitable(6)resource allocation in other contexts (19–22). However, the public healthcare system covering the uninsured population in Mexico has simply quantified each individual resource (i.e. number of CT scan per region, number of specialists) but has not geographically mapped resources considering all the patient needs across the cancer continuum or journey(i.e. laboratory, CT scan, nurses, oncologists, pathologist, nutritionist, etc) nor has it cross-mapped resources with population density .
		As part of the project(18), this analysis employed a cross-sectional study design to assess the available diagnostic and therapeutic infrastructure for five most relevant cancer types in Mexico: breast, lung, prostate, colorectal and cervix (18). These site specific cancers were chosen due to high incidence-mortality-ratios and impact as catastrophic expenditures (7,19–21). A realist framework informed the analysis, drawing on existing literature on cancer infrastructure assessment (22–28).<br>Relevant datasets were selected based on their potential to provide comprehensive information on oncological infrastructure, including facilities, equipment, and services for the public healthcare facilities for non-insured patients. These were accessible through official health-related websites in Mexico i.e. from the General Direction of Health Information (DGIS), Health Sectorial Resources 2022 and from the National Centre of Excellence Technology in Health (CENETEC-Salud), the High Specialty Medical Equipment National Inventory (EMAT) 2016 (29,30).<br>Facilities in Mexico were identified through a unique identifying number (CLUES) according to official records at DGIS databases. Datasets were merged and duplicate facilities were deleted. To assess regional disparities, the information extracted from the database categories was mapped to identify which municipalities held available infrastructure and was cross mapped with municipal population density to provide visual aid on the geographical distribution of facilities by population.<br>The development of the categorization scheme for the infrastructure and human resources for cancer was based on the National Comprehensive Cancer Network guidelines. However, to ensure the accuracy, appropriateness for the Mexican context, and consider the available data, systematic validation process was implemented, involving pilot testing and cross validation with expert panel, consulting with six domain experts in oncology and public health in Mexico. As a result, two infrastructure categories arose from expert consensus: facilities that can suspect cancer and provide a preliminary diagnosis and facilities that confirm	Thank you. We have spit the methods section and ensured the text was easier to read. We do not think	Changed	**Study design**<br>As part of the BLINDED project(19), five site specific cancers were chosen for this cross sectional study, due to high incidence-mortality-ratios and impact as catastrophic expenditures (6,24–26). This analysis identified the available diagnostic and therapeutic infrastructure for five major cancer types in Mexico: breast, lung, prostate, colorectal, and cervical cancers (23).
				Changed	Data source and eligibility<br>Publicly available datasets were sought and selected based on their potential to provide comprehensive information on oncological infrastructure, including facilities, equipment, and services for the public healthcare facilities for non-insured patients. These were accessible through official health-related websites in Mexico i.e. from the General Direction of Health Information (DGIS), Health Sectorial Resources 2022 and from the National Centre of Excellence Technology in Health (CENETEC-Salud), the High Specialty Medical Equipment National Inventory (EMAT) 2016 (34,35). Facilities in Mexico were identified through a unique identifying number (CLUES). Datasets were merged and complemented, in the case of duplicaton of resources the most recent resources were considered for the analysis .
					**Data analysis** Six domain experts in oncology and public health in Mexico were involved in initially identifying the diagnostic and therapeutic resources needed by each cancer type. Infrastructure and human resources were categorized and selected per cancer based on existing ASCO Guidelines (30–36) and on the National Comprehensive Cancer Network Guidelines for resource stratification (37). However, to ensure that these were categorised accurately for the Mexican context, consensus was made between the experts to develop a classification based on the available data. As a result, from the merged resources database, two resource categories arose from consensus: service scope (diagnostic or diagnostic and therapeutic) and infrastructure level (core or enhanced). Table 1 shows the resources available from each dataset and how each one was classified in both service scope and infrastructure level for each cancer type. If deemed unnecesary for the cancer type in question, the cell was coloured in gray. The designation “INDISP” was used to classify each resource when they were indispensable for core patient care and alternatively, the “EXPEND” designation was used when, resources would enhance patient care. The rationale for this “EXPEND” was to include facilities that may provide enhanced cancer care through physical therapy, nutritionists or other infrastructure alternatives in addition to diagnosis or treatment.

REVIEWER 3	Methods are not clear. Need to more clearly plit in sections with informative subtitles and describe in detail each step. Maybe also a figure that explains step by step what was done.	diagnosis and stage, identifies cancer subtypes, and provides treatment.<br>Additionally, the designation "INDISP" was used to classify resources indispensable for a core diagnosis and treatment of a patient. Alternatively, the "EXPEND" designation represents elements that, following a patient-centred logic, would be required for enhanced care of the patient; however, their availability did not hinder management or impair diagnosis and treatment. Table 1 describes the infrastructure typology developed and Table 2 shows the resources necessary for preliminary diagnosis or diagnosis and treatment and the resources used to classify health facilities into core or enhanced.<br>To avoid over-exclusion, we did not consider general practitioners as an exclusion criterion for facilities in the complete pathway category. It was assumed that in most specialised hospitals, which focus on specific diseases like cancer, would not typically employ general practitioners, instead, these hospitals would rely on specialists and subspecialists for patient care.<br>For the initial stage of care (up to the point of suspicion of cancer), either a general practitioner or a specialist (urologist, gynaecologist, gastroenterologist or pneumologist) was considered sufficient, and thus both were given equal weight (coded as 1). However, for the facilities providing both diagnosis and treatment, it was necessary to have a general medical oncologist in the facility, while other specialists were optional.<br>When a specific imaging technique such as ultrasound was required for diagnosis (but unavailable), higher-technology alternatives like tomography (CT or MRI scans) were used to categorise the facility instead. This ensured that our classification was not overly strict. For instance, some breast or colon cancer diagnostic procedures could potentially be done using ultrasound but, if unavailable, they can be done using CT instead. Thus, the facility would be marked as "available".<br>Once consensus was reached over the classification of resources, maps were drawn. Two types of maps were developed: 1) maps showing facilities providing core cancer care and 2) maps showing facilities providing enhanced care (Supplementary Maps 2A-E). The rationale for this secondary analysis was to include units that may provide enhanced cancer care through physical therapy, nutritionists or other infrastructure alternatives for diagnosis or treatment.	its necessary to add more figures, but we have moved the tables around and ensured the text desrcibes the tables content and ensured its location in the document is appropiate.	Changed	Data validation and mapping resources: Once consensus was reached over the classification of resources, facilities were judged in service scope and infrastructure level. In order to be classified into these accordingly, all resources from Table 1 marked in each column as necessary had to be available in the database. As a result of this exercise, facilities were mapped per cancer type according to service scope (diagnostic or diagnostic and therapeutic) and infrastructure level (core or enhanced). As a result of this exercise, facilities were mapped per cancer type according to service scope (diagnostic or diagnostic and therapeutic) and infrastructure level (core or enhanced). The facilities were mapped against municipal population density to provide visual aid on the geographical distribution of facilities by population.Table 2 describes the two types of maps developed: maps showing facilities providing core cancer care (Fig. 1a-e) and maps showing facilities providing enhanced care (Fig 2a-e).<br>A systematic validation of maps was conducted, involving the revision of a random sample of 25% of the facilities coded resources, and facility verification with the expert panel (i.e. when facilities were known by experts to have oncological resources but did not appear in the map, the team verified the resources preventing the facility to be visible in the map), without finding any misclassifications in either the random sample or the manually searched facilities.<br><br>Up to the point of cancer suspicion, facilities with either a general practitioner or a specialist (urologist, gynaecologist, gastroenterologist or pneumologist) was considered to be sufficient for diagnostic service scope. However, for the facilities providing both diagnosis and treatment, it was necessary to have a general medical oncologist in the facility to be included in the map, without the necessity of an existing general practitioner. Thus, to avoid over-exclusion of facilities in the maps, we did not consider general practitioners as an exclusion criterion for facilities marked as diagnostic and therapeutic. It was assumed that in most specialised hospitals providing treatment, which focus on specific diseases like cancer, would not typically employ general practitioners, instead, these hospitals would rely on specialists and subspecialists for patient care.<br><br>When a specific imaging technique such as ultrasound was required for diagnosis (but unavailable), higher-technology alternatives like tomography (CT or MRI scans) were used to categorise the facility instead. This ensured that our classification was not overly strict. For instance, some breast or colon cancer diagnostic procedures could potentially be done using ultrasound but, if unavailable, they can be done using CT instead. Thus, in such cases, the facility was marked as holding the resource if either one was available.
	What is it meant with "most relevant cancers"?	As part of the xxx project(18), this analysis employed a cross-sectional study design to assess the available diagnostic and therapeutic infrastructure for five **most relevant cancer types** in Mexico: breast, lung, prostate, colorectal and cervix (18). These site specific cancers were chosen due to high incidence-mortality-ratios and impact as catastrophic expenditures (7,19–21). **A realist framework informed the analysis**, drawing on existing literature on cancer infrastructure assessment	We have modified the sentence so it is clear why we chose the 5 types of cancer. This can also be found in the reference suggested.	Changed	As part of the BLINDED project(23), five site specific cancers were chosen for this cross sectional study, due to high incidence-mortality-ratios and impact as catastrophic expenditures (6,24–26). This analysis identified the available diagnostic and therapeutic infrastructure for five major cancer types in Mexico: breast, lung, prostate, colorectal, and cervical cancers (23).
	What is a "realist framework"?		We changed the wording to avoid readers to think it is a framework.	Changed	Data analysis Six domain experts in oncology and public health in Mexico were involved in initially identifying the diagnostic and therapeutic resources needed by each cancer type. Infrastructure and human resources were categorized and selected per cancer based on existing ASCO Guidelines (30–36) and on the National Comprehensive Cancer Network Guidelines for resource stratification (37). However, to ensure that these were categorised accurately for the Mexican context, consensus was made between the experts to develop a classification based on the available data. As a result, from the merged resources database, two resource categories arose from consensus: service scope (diagnostic or diagnostic and therapeutic) and infrastructure level (core or enhanced). Table 1 shows the resources available from each dataset and how each one was classified in both service scope and infrastructure level for each cancer type. If deemed unnecesary for the cancer type in question, the cell was coloured in gray. The designation "INDISP" was used to classify each resource when they were indispensable for core patient care and alternatively, the "EXPEND" designation was used when, resources would enhance patient care. The rationale for this "EXPEND" was to include facilities that may provide enhanced cancer care through physical therapy, nutritionists or other infrastructure alternatives in addition to diagnosis or treatment.
	Methods. Paragraph 1, last sentence "drawing on existing literature on cancer infrastructute assessment (22-28)". I don't see the infrastructure assessment in the cited documents, these are ASCO resource-stratified clinical managment guidelines for specific cancer sites. Not clear how these were used to assess infrastructure. Please explain.	Al Sukhun S, Temin S, Barrios CH, Antone NZ, Guerra YC, Chavez-MacGregor M, et al. Systemic Treatment of Patients With Metastatic Breast Cancer: ASCO Resource-Stratified Guideline. JCO Glob Oncol. 2024 Jan;10:e2300285.<br>23. Shastri SS, Temin S, Almonte M, Basu P, Campos NG, Gravitt PE, et al. Secondary Prevention of Cervical Cancer: ASCO Resource-Stratified Guideline Update. JCO Glob Oncol. 2022 Sep;8:e2200217.<br>24. Chuang LT, Temin S, Berek JS, Management and Care of Patients with Invasive Cervical Cancer Resource-Stratified Guideline Expert Panel. Management and Care of Patients With Invasive Cervical Cancer: ASCO Resource-Stratified Guideline Rapid Recommendation Update. JCO Glob Oncol. 2022 Mar;8: e2200027.<br>25. Chiorean EG, Nandakumar G, Fadelu T, Temin S, Alarcon-Rozas AE, Bejarano S, et al. Treatment of Patients With Late-Stage Colorectal Cancer: ASCO Resource-Stratified Guideline. JCO Glob Oncol. 2020 Mar;6:414–38.<br>26. Lopes G, Stern MC, Temin S, Sharara AI, Cervantes A, Costas-Chavarri A, et al. Early Detection for Colorectal Cancer: ASCO Resource-Stratified Guideline. J Glob Oncol. 2019 Feb;5:1–22.<br>27. Arrossi S, Temin S, Garland S, Eckert LO, Bhatla N, Castellsagué X, et al. Primary Prevention of Cervical Cancer: American Society of Clinical Oncology Resource-Stratified Guideline. J Glob Oncol. 2017 Oct;3(5):611–34.<br>28. Shaw SE, Paparini S, Murdoch J, Green J, Greenhalgh T, Hanckel B, et al. TRIPLE C reporting principles for case study evaluations of the role of context in complex interventions. BMC Med Res Methodol. 2023 May 13;23(1):115.	We used the available literature to draw on our resource classification. We also made to include the reference of the NCCN in the references and not only in the text. We changed some of the original text and made sure to include more detail.	Changed	
	a. Please specify the consulted databases. What is it meant by "relevant datasets"?	Relevant datasets were selected based on their potential to provide comprehensive information on oncological infrastructure, including facilities, equipment, and services for the public healthcare facilities for non-insured patients.	Methods section was revised, edited and provided with more details.	Changed	Publicly available datasets were sought and selected based on their potential to provide comprehensive information on oncological infrastructure, including facilities, equipment, and services for the public healthcare facilities for non-insured patients. These were accessible through official health-related websites in Mexico i.e. from the General Direction of Health Information (DGIS), Health Sectorial Resources 2022 and from the National Centre of Excellence Technology in Health (CENETEC-Salud), the High Specialty Medical Equipment National Inventory (EMAT) 2016 (34,35). Facilities in Mexico were identified through a unique identifying number (CLUES). Datasets were merged and complemented, in the case of duplicaton of resources the most recent resources were considered for the analysis. The source of each resource considered is available in the first column of Table 1.
	b. What were the specific variables used?	Table 4	Variables were already listed but now they are listed in the Table1, including the codes of the variables. See table 1 Additionally, the text mentions where the variables chosen and codes can be found.	Partially Changed	
	c. What is it meant with "database categories" used for "mapping"?		This text now described how categories were built and what they are: service scope and infrastructure level. We changed some of the original text and made sure to include more detail.	Changed	**Data analysis**: Six domain experts in oncology and public health in Mexico were involved in initially identifying the diagnostic and therapeutic resources needed by each cancer type. Infrastructure and human resources were categorized and selected per cancer based on existing ASCO Guidelines (30–36) and on the National Comprehensive Cancer Network Guidelines for resource stratification (37). However, to ensure that these were categorised accurately for the Mexican context, consensus was made between the experts to develop a classification based on the available data. As a result, from the merged resources database, two resource categories arose from consensus: service scope

d. What is it meant by "development of the categorization scheme"?	.**The development of the categorization scheme** for the infrastructure and human resources for cancer was based on the National Comprehensive Cancer Network guidelines. However, to ensure the accuracy, appropriateness for the Mexican context, and consider the available data, **systematic validation process was implemented**, involving pilot testing and cross validation with expert panel, consulting with six domain experts in oncology and public health in Mexico. As a result, two infrastructure categories arose from expert consensus: facilities that can suspect cancer and provide a preliminary diagnosis and facilities that confirm diagnosis and stage, identifies cancer subtypes, and provides treatment.	We have now described more thoroughly what the process was to build these categories, what we based ourselves in and the specific considerations for each category. We changed some of the original text and made sure to include more detail.	Changed	(diagnostic or diagnostic and therapeutic) and infrastructure level (core or enhanced). Table 1 shows the resources available from each dataset and how each one was classified in both service scope and infrastructure level for each cancer type. If deemed unnecesary for the cancer type in question, the cell was coloured in gray. The designation "INDISP" was used to classify each resource when they were indispensable for core patient care and alternatively, the "EXPEND" designation was used when, resources would enhance patient care. The rationale for this "EXPEND" was to include facilities that may provide enhanced cancer care through physical therapy, nutritionists or other infrastructure alternatives in addition to diagnosis or treatment.
e. What was the "systematic valifation process" that took place? What did the pilot testing consist of?		the validation process consisted of revision of sample and verifying specific facilities resources. We developed maps and then the experts would ask us about the ones they thought were missing and we verified if we had made any mistakes. 100% were not mistakes. Facilities did not have the complete list of resources chosen by the expert panel to be visible in the map. We changed some of the original text and made sure to include more detail.	Changed	A systematic validation of maps was conducted, involving the revision of a random sample of 25% of the facilities coded resources, and facility verification with the expert panel (i.e. when facilities were known by experts to have oncological resources but did not appear in the map, the team verified the resources preventing the facility to be visible in the map), without finding any misclassifications in either the random sample or the manually searched facilities.
Not sure ref 7 is the best to support the description of the Mexican healthcare system.	In 2022, Mexico allocated 5.5% of its GDP to healthcare, which is below the Organisation for Economic Co-operation and Development (OECD) average(1). The number of hospital beds per 1000 inhabitants in Mexico is also significantly lower than the OECD average of 4.8, standing at a mere 1.5 (2–6). Consequently, the gap between supply and demand creates major challenges for Mexico in achieving optimal health outcomes (7).	We have ensured our references were in the correct order and represented the correct bibliography to describe the Mexican Health care system	Changed	--- Gómez-Dantés O, Sesma S, Becerril VM,Knaul FM, Arreola H, Frenk J. Sistema de salud de México. Salud Publica Mex. 2011;53(2):S220–32.<br>----Goss PE, Lee BL, Badovinac-Crnjevic T, Strasser-Weippl K, Chavarri-Guerra Y, St Louis J, et al. Planning cancer control in Latin America and the Caribbean. Lancet Oncol. 2013 Apr;14(5):391–436.<br>---- Knaul FM, Arreola-Ornelas H, Touchton M, McDonald T, Blofield M, Avila Burgos L, et al. Setbacks in the quest for universal health coverage in Mexico: polarised politics, policy upheaval, and pandemic disruption. Lancet. 2023 Aug 26;402(10403):731–46.
Results. Suggestion: next to each number of facilities per cancer type provide in parenthesis the percentage out of the 686 core cancer facilities, instead of reporting numbers in this sentence and then percentages in the next 2 sentences.	From the original national database, a total of 14133 unique healthcare facilities that deliver any type of healthcare were identified, out of which N=686 (5%) provided core cancer care and N=404 (3%) provided enhanced cancer care.<br>Core capacity and capability up to preliminary diagnosis for colorectal cancer was present in 614 facilities, N=157 for breast, N=479 for cervical, N=64 for lung, and N=104 for prostate. In contrast, core facilities that provide diagnosis and treatment include 14 treatment centres that had the workforce and infrastructure to deliver colorectal cancer care, N=9 breast, N=8 cervical, N=13 lung, and N=8 prostate. Table 3 shows that from the 686 unique facilities, 92% provided core care to colon cancer, 71% to cervical cancer, 24% to breast cancer, 16% to prostate cancer and 11% lung cancer. However, more than 70% of the facilities providing care for breast, cervical, lung and prostate had enhanced infrastructure whereas 57% of colon cancer had enhanced care. Figures 1A-E map facilities classified according to the core care criteria chosen by the expert panel over the population density per municipality. These maps show which facilities can provide core cancer care up to diagnostic suspicion compared to which facilities can provide a complete pathway to cancer diagnosis and treatment. Similarly, supplementary Figures 2A-E map the enhanced cancer care facilities per cancer type.<br>Cancer specific core or enhanced infrastructure that can provide diagnosis and radiotherapy or surgery at the national level included N=10 brachytherapy units distributed in 10 facilities, N=11 cobalt-60 in 11 facilities, N=21 linear accelerators in 14 facilities, and N=188 operating rooms distributed across 14 facilities. The distribution of therapeutic infrastructure for cancer diagnosis and treatment is visible in Figure 3. Lastly, Table 4 shows the ratio of health oncologists per cancer care facility is 4.7.	There might be facilities that have core resources for all cancer types or just some. Therefore facilities can become duplicated. That is why they are reported individually in absolut numbers and not in percentages. The percentage of the facilities with the 686 as numerator calculates the relative number in accordance to the total number of facilities in cancer care for each cancer individually. We have made changes to Table 3 for the numbers to be clear and more easily understood by the reader. Additionally, we changed the text so it is consistent with the numbers.. even if its is more duplicative to avoid any confusion. We also added some labels to facilitate the identification of resources being described.	Changed	From the original national database, a total of 14133 unique healthcare facilities that deliver any type of healthcare were identified, out of which N=686 (5%) provided cancer care. Table 3 shows the N=686 facilities found stratified by infrastructure level (core or enhanced) and service scope (diagnostic or diagnostic and therapeutic) split by cancer type.<br>**Faciltiies providing core cancer care**<br>Out of the N=686 facilities providing some type of cancer care, for colon cancer particularly N=628 provided core care (92%). However, only N=14 of those core facilities provided diagnosis and treatment, whilst the rest (N=614) only provided diagnosis. This indicates a significant gap in the availability of core therapeutic options for colon cancer patients. The other types of cancer show similar core infrastructure gaps, cervical cancer facilities totaled 487 facilities (71%), with 479 focusing solely on diagnosis and 8 offering treatment. Accounting for 24% of the total number of cancer facilities, 166 facilities were identified for breast cancer, with 157 offering diagnostic services and only 9 providing both diagnosis and therapeutic care. Lung cancer facilities numbered 77 (11%), with 64 providing diagnostic services and only 13 offering both core diagnosis and treatment. Prostate cancer facilities totaled 112 (16%), with 104 focused on diagnostic services and only 8 providing both. Figures 1A-E map facilities classified according to the core care criteria chosen by the expert panel over the population density per municipality. These maps show which facilities can provide core cancer care up to diagnostic suspicion compared to which facilities can provide a complete pathway to cancer diagnosis and treatment.<br><br>**Faciltiies providing enhanced cancer care**<br>From the N=14133 facilities providing some type of care, and the N=686 providing some type of cancer care, only N=404 provided enhanced cancer care. Thus, even less number of infrastructure is found to be enhanced. Breast, prostate and lung cancer care are the ones that least benefit from care provided in an enhanced environment. Figures 2A-E map the enhanced cancer care facilities per cancer type.<br>Although colon cancer shows high levels of availability of core facilities compared to other cancer types, it also has the highest difference in core and enhanced infrastructure, showing almost half of the facilities (43%) are not enhanced. In contrast, almost all lung cancer facilities that are core also classify as enhaced infrastructure, with only a 5% difference.<br>**Advanced infrastructure and human resources**<br>Cancer specific core or enhanced infrastructure that can provide diagnosis and radiotherapy or surgery at the national level included N=10 brachytherapy units distributed in 10 facilities, N=11 cobalt-60 in 11 facilities, N=21 linear accelerators in 14 facilities, and N=188 operating rooms distributed across 14 facilities. The distribution of therapeutic infrastructure for cancer diagnosis and treatment is visible in Figure 3. Lastly, Table 4 shows the ratio of health oncologists per cancer care facility is 4.7.
Results. I wonder if there are key missing data from the datasets obtained, as I don't see cancer services available for the uninsured that I know exist in Jalisco (Guadalajara) and in Nuevo León (Monterrey).	Section 5 of Discussion>	In the methods section we added further description of how we validated data and made sure that our coding was not wrong. There were several cases in which the expert panel wanted to check why specific facilities were not visible. It was always the case that they were missing some of the elements to be classified into the different categories.	Changed	A systematic validation of maps was conducted, involving the revision of a random sample of 25% of the facilities coded resources, and facility verification with the expert panel (i.e. when facilities were known by experts to have oncological resources but did not appear in the map, the team verified the resources preventing the facility to be visible in the map), without finding any misclassifications in either the random sample or the manually searched facilities.

		Section 5 of Discussion> The lack of precise and real-time data on the facilities' available infrastructure and functioning equipment makes the patient journey very complex. Thus, navigating the patient into the appropriate health facility is a hard task. Precise and updated information should be available to facilitate patient navigation in real time.................<br><br>Section 5 of Discussion> The CENETEC database was last updated in 2016 and DGIS has not updated the database since 2022. Thus, these maps might not account for changes in infrastructure that happened after. Moreover, the DGIS does not routinely collect data from the private hospitals regarding their infrastructure. Thus, these maps do not show the private health-care facilities that might provide care to patients.<br><br>Section 5 of Discussion> Another limitation stems from the insufficient specificity of available information regarding whether the facilities meet the criteria and possess all the necessary resources for treating specific types of cancer. This issue is of paramount importance, given the common misconception that cancer is a singular disease when, each cancer type necessitates highly specific elements for accurate diagnosis and treatment.	This is mentioned in the discussion in Section 5. It is part of the major recommendations we make to the mexican goverment. They should keep these updated and alive in order to have a proper view of what is still there and what isnt.	Not changed	No changes
	17. Tables<br>- Not clear to me what tables correspond with those cited in the manuscript. Please name the files according to the table referred to in the text.<br>- Tables 41 and 42 are uncomprehensible to me.		We have made sure the numbering is clear in the text and the tables/images. Additionally, tables were replaced in the manuscript so that it more accurately portrayed the step in the process it was describing. Thank you for the recommendation	Changed	Changes in figures and tables numbering and order in text appearance.
	Discussion Again not clear why this was a "patient-centered approach to map resources"?.	Geolocalisation of healthcare resources has proven to facilitate policy-making for health resource allocation (14–17). Hence, a patient-centred geographical assessment of the human resources and infrastructure allocated for each cancer type is necessary for healthcare planning and achieving an equitable distribution of resources(7).	We have deleted this from the manuscript to avoid confusion and rephrased in the justification of the project to what our aim was. We have also provided more examples to make it more clear.	Changed	Mapping healthcare resources (or the lack off) has proven to facilitate policy-making for more equitable(6)resource allocation in other contexts (19–22). However, the public healthcare system covering the uninsured population in Mexico has simply quantified each individual resource (i.e. number of CT scan per region, number of specialists) but has not geographically mapped resourc considering all the patient needs across the cancer continuum or journey(i.e. laboratory, CT scan, nurses, oncologists, pathologist, nutritionist, etc) nor has it cross-mapped resources with populatio density.
	Discussion In general, it seems that centers are located in more densly populated areas, which woudl suggest that this was considered in health policy planning?	Resources are not necessarily allocated in areas where population density seems higher. Disparities in resource allocation across municipalities can lead to inequalities in access to detection, early diagnosis, and treatment, as well as worsened outcomes and increased costs. The unequal distribution of health infrastructure not only negatively affects health outcomes through the lack of access but also by the inability of different regions to train human resources(7). Addressing these challenges requires a comprehensive approach that involves strengthening diagnostic capacities for cancer at the primary and secondary care levels(7,40,41). More research should be done to understand the factors behind resource allocation, infrastructure, cancer incidence, and human development index to see if there is an association between the variables.	Our data suggests that although a few state capital cities have units that fulfill core and enhanced resources needed for some types of cancers (Monterrey, Guadalajara, etc.), most of other urban areas in the country are not covered for complete treatment pathway on the patient journey. Take for example breast cancer, where only 9 of the 31 states of Mexico have units that fulfill all recommended enhanced resources (or treatment) at least on one urban area. This confirms the current disparity on access for oncology-related healthcare in our country, by lacking equitable policy planning.	Not changed	No changes
	Discussion Add in limitations (data available) the fact that there seem to be services available missing in some key metropolitan cities (Guadalajara, Monterrey, others?)	Section 5 of Discussion> The lack of precise and real-time data on the facilities' available infrastructure and functioning equipment makes the patient journey very complex. Thus, navigating the patient into the appropriate health facility is a hard task. Precise and updated information should be available to facilitate patient navigation in real time.................<br>Section 5 of Discussion> The CENETEC database was last updated in 2016 and DGIS has not updated the database since 2022. Thus, these maps might not account for changes in infrastructure that happened after. Moreover, the DGIS does not routinely collect data from the private hospitals regarding their infrastructure. Thus, these maps do not show the private health-care facilities that might provide care to patients.<br>.Another limitation stems from the insufficient specificity of available information regarding whether the facilities meet the criteria and possess all the necessary resources for treating specific types of cancer. This issue is of paramount importance, given the common misconception that cancer is a singular disease when, each cancer type necessitates highly specific elements for accurate diagnosis and treatment.	All of these were addressed already in discussion Section 5: Obtaining precise and updated infrastructure data	Not changed	No changed

Comment	Response
1. Please provide a title page that includes the names (authorship list), affiliated institutions with countries and full postal addresses. Also provide the email address for the submitting/corresponding author.	Done
2. As mentioned before, please edit the title for clarity, i.e. please remove the text “A Call for Policy Reform and Infrastructure Enhancement to Enable Equitable Cancer Care”. Furthermore, please update the remaining title to say “Assessing Disparities in Cancer Care Resource Distribution in Mexico”.	Done
3. Please ensure that the Abstract contains the following sections: Background, Methods, Results, Conclusions.	Done
4. Please consider elaborating more on the 5 key points highlighted in the abstract results section, however remember that the maximum word limit for the abstract section is 350.	Done
5. We recommend editing the main text for English language and grammar to improve readability and clarity for our readers. Please proofread the manuscript.	Done
6. Please provide a brief introduction into cancer generally into the Background section.	Done
7. Please use abbreviations throughout the manuscript once defined. For example, define GDP.	Done
8. When referring to ‘cancer resource’, please rephrase it to say ‘cancer care resources’.	Done
9. In the third paragraph of the background section, we would prefer that when referring to counts of populations that whole numbers, rather than numbers with decimal points, are used for clarity. E.g. the number of oncology surgeons and paediatric oncologist surgeons per state were around one per million inhabitants, and the distribution of oncologists and gynaecologist-oncologists were sometimes even less so.	I think this paragraph comes out of an article and it reflects even less covarge than rounding up the numbers.
10. In the first sentence of the Methods, please provide a URL for the BLINDED project, if possible. Please provide more details about this project.	The project is already there as part of the references

11. References 6 and 27 are used to support a statement about justifications for selecting the cancer sites based on incidence-mortality ratios and impact as catastrophic expenditures, however the evidence from these citations are 10+ years old. Is it possible to provide more recent supportive evidence?	In fact 6, 25-27 are used and they are specifically mentioning Mexico.We have added other sources that are either cancer specific or regional that support this.
12. Please also provide relevant references for the analytical mapping, if possible.	We have added a figure to describe the analytical process. This is linked to the question regarding realist framework
13. Please provide URLs to any data sources that are used, if possible.	URL are already in the references
14. Please clarify in the Data source and eligibility section if "CLUES" is an abbreviation - if so, please define it first before use. If not, please provide further detail as to what "CLUES" is. Furthermore, please correct the spelling of "duplication" in the following sentence.	Done
15. Please clarify in the Methods section who the 6 Domain experts are?	Done
16. Please ensure that all methodological approaches and decisions are clarified.	We have added a figure to describe the analytical process.
17. In a previous version of the manuscript, a realist framework was mentioned - Please can you clarify what this is and why this is not included in the current version of the manuscript? Essential methods details need to be provided to ensure transparent reporting and reproducibility.	In our previous manuscript it was deleted as it created confusion in the reviewers. This time we have brought the term back an included a single paragraph describing the framework: context, mechanisms and outcome.
18. Please clarify in the first sentence of the Results section which database was used, and if more were used, please also provide details for these.	Our database, the one previously described as merged in the methods section. We changed the wording so its clear now.
19. Please remove the numbered subheadings throughout the Discussion section.	Done
20. Please clarify in the Discussion what "linear accelerator" means? This is unclear and needs defining in the Methods. Infrastructure types were not introduced in the Methods.	Done
21. Please relabel any reference in the text to "Supplementary Material" as "Additional File X", depending on the number of the Additional File.	Done

22. The additional file is mentioned only in the Discussion, but the information within the file has contributed to the results of the study, so please present the additional file in the Results section as well. Furthermore, please elaborate on the details of the file further to enable a better understanding for the reader. Furthermore, it's not clear where the "recommendations" are in the file as to the more precise classifications of infrastructure and resources and how these were derived. We would prefer that any recommendations are not presented as it currently remains unclear how they were derived.	The additional file was relabeled as "Recommendations for further improvement in data collection". We believe this document is important for DGIS and CENETEC to update their data collection codes. We have made the exercise of marking what needs to be added or changed. This is not part of the results section, but a set of general recommendations in accordance to our findings. Once this document is published, there will be a meeting with the DGIS to go through each one of these recommendations and a panel will be made to generate a final list of ancer infrastructure codes and subcodes.
23. Please edit the Discussion significantly to only provide discussion of your findings in the context of literature, with limited speculation, and feed this discussion into the justification for recommendations that you provide.	We added references and modified slightly to avoid speculation. Additional file B is part of the discussion, as it is a set of recommendations that are discussed throughout the discussion section, but doest not need to be integrated into the main manuscript. It is used only as a reference of all the things that should/could be changed
24. Please edit the paragraph in the Discussion section starting with "[BLINDED]"for readability.	Unblinded and started sentence with project name
25. Please provide a reference for the CENETEC database and DGIS in the Discussion section.	Done
26. Please rename 'Conclusion' as 'Conclusions'.	Done
27. Please consider condensing the conclusion section; this will help in delivering the key messages from the study. Please also consider removing the first sentence about the aims, we do not feel these need to be restated.	Done

28. Please include a Declarations section and all the subheadings listed on our website at https://bmcglobalpublichealth.biomedcentral.com/submission-guidelines/preparing-your-manuscript/research at the end of the main text in your manuscript, including Ethics approval and consent to participate, Consent for publication, Competing interests, Acknowledgements, Funding, Author contributions, and Availability of data and materials. If sections do not apply, please state 'not applicable'.	Done
29. Please include a statement in the Ethics approval and consent to participate subsection of the Declarations section describing the ethics approval and informed consent obtained for this study, including the name of the ethics committee that approved the study and the committee's reference number if appropriate. If ethics approval was waived, please explain the reasons for this.	Done
30. Please provide a data statement in the Availability of data and materials section of the declarations (and update the data statement on the editorial system). In accordance with our policies, we require that all datasets and code on which the conclusions of the paper rely are deposited in publicly available repositories according to community standards, where compliant with ethics approvals, patient consent and confidentiality agreements, or are provided within the manuscript and its supporting files. Please note that data access on reasonable request is not acceptable at our journals. Non-compliance must be justified in full and this will be reviewed by the editorial team. Please ensure that this is addressed prior to resubmission. If this is not possible, please provide your data-sharing timeline. Please see Springer Nature's information on recommended repositories https://www.springernature.com/gp/authors/research-data-policy/recommended-repositories.	Done
31. In the Competing interests section of the declarations, please provide a competing interests declaration from all authors detailing any competing interests; if you do not have any competing interests, please state "The authors declare that they have no competing interests" in this section. Please see our guidelines for further information.	Done

32. Please declare all sources of funding for the reported research in the funding section of the declarations. If the funder has a specific role in the conceptualization, design, data collection, analysis, decision to publish, or preparation of the manuscript, this should be declared.	Done
33. The individual contributions of authors to the manuscript should be specified in the Authors contribution section. Please also ensure this section includes the text "All authors read and approved the final manuscript".	Done
34. In the Acknowledgements section of the declarations, please acknowledge anyone who contributed towards the article who does not meet the criteria for authorship including anyone who provided professional writing services or materials. If you do not have anyone to acknowledge, please write "Not applicable" in this section.	Done
35. Please upload all main figures as figure files, and place figure legends separately after the references section. If any updates are needed for figures, please make necessary changes.	Done
36. As per point 34, please upload each figure as a combined single figure, not separate pieces - potentially consider using Powerpoint for large figures.	We have donde this only for Table 1. (Table 1 is a table in a figure format)
37. We noticed that figures/maps have a note below them saying "Sourse (spelling) Own elaboration." Unless any figures/maps were not generated from the results of this study, then please remove these notes. Please clarify this.	We have removed the notes
38. Please make sure that figures are cited in ascending order.	Done
39. Please note that main manuscript tables larger than one page A4 need to be provided at the end of the manuscript, e.g. Table 1 should be moved.	Done

40. Please clarify what expend means in Table 1 and ensure all abbreviations are defined and a legend is provided.	Previously the expend legeand was in the image. This was later changed due to comments from previous reviewers. Currectly, it is in the methods section >> **The designation "INDISP" was used to classify each resource when they were indispensable for core patient care and alternatively, the "EXPEND" designation was used when resources would enhance patient care. The rationale for this "EXPEND" was to include facilities that may provide enhanced cancer care through physical therapy, nutritionists or other infrastructure alternatives in addition to diagnosis or treatment.**
41. Please remove main figure legends from the figure files and only provide after the Declarations section.	Done
42. Please rename the supplementary file as 'Additional file X', and cite explicitly by additional file name in the manuscript e.g. 'Additional file 1: Fig. S1' or 'Additional file 1: Table S1. Please ensure that if you have more than one additional file that they are cited in ascending order within the main body of text.	We decided to name them :<br>**Additional File A:** Analytical process for classification of scope and infrastructure level<br>**Additional File B:** Recommendations for further improvement in data collection
43. Within the additional file document, please provide a main heading "Additional File 1".	
44. Please provide a title for the table in Additional File 1 as "Table S1: Assessment of codes" - or something similar.	
45. Please provide a subsection after the declarations section listing all the additional files including file names (e.g. Additional file 1), titles and a short description of data.	
46. Please ensure that you update your reference list in case any of the potentially included preprint manuscripts have been published in peer-reviewed journals in the meantime.	Done

47. We request that you let us know upon resubmission whether your manuscript will have an institutional press release. It is important that you initiate the steps for potential press releases at this stage in order to ensure that we can accommodate embargo dates for your manuscript. Additionally, we encourage you to highlight your work on the Springer Nature Research Communities webpage, specifically the Public Health section. Please see here for more details https://communities.springernature.com/ and contact communities@springernature.com.	Until now there is no current press release associated with the document. This is usually done after the document is published in Mexico.
48. Please provide a point-by-point response to all requests and please upload a clean version of the manuscript as the main manuscript file and clean versions of all additional files. If possible, a marked up version does not need to be uploaded but should instead be sent via email to bmcglobalpublichealth@biomedcentral.com.	Done
